# Contractile effects of dulaglutide in the human atrium

**DOI:** 10.1007/s00210-026-05647-5

**Published:** 2026-07-01

**Authors:** Joachim Neumann, Milena Jarikova, Britt Hofmann, Uwe Kirchhefer, Ulrich Gergs

**Affiliations:** 1 Institute for Pharmacology and Toxicology, Medical Faculty, Martin-Luther-University Halle-Wittenberg, Magdeburger Straße 4, 06097 Halle (Saale), Germany; 2 Department of Cardiac Surgery, Mid-German Heart Centre, University Hospital Halle, Ernst-Grube-Straße 40, 06097 Halle (Saale), Germany; 3 Institute for Pharmacology and Toxicology, Medical Faculty, Münster University, Domagkstraße 12, 48149 Münster, Germany

**Keywords:** Dulaglutide, Human atrium, Inotropy

## Abstract

Dulaglutide is a glucagon-like peptide 1 receptor (GLP-1R) agonist. Dulaglutide (LY2189265) is a GLP-1(7–37) analogue fused by a linker to a modified immunoglobulin G. GLP-1R agonists like dulaglutide can be used to treat diabetes type 2 and obesity. We tested the hypothesis that dulaglutide directly increased force of contraction in the human heart via GLP-1R. To this end, we conducted contraction experiments in paced (1 Hz) isolated human right atrial muscle preparations (HAP). HAP were obtained during open-heart surgery from adult patients with severe coronary heart disease. We detected a concentration- and time-dependent positive inotropic effect of dulaglutide in HAP. Dulaglutide augmented the rate of tension development, the rate of tension relaxation and accelerated the relaxation time in HAP. Dulaglutide (100 nM) augmented the phosphorylation state of phospholamban at serine 16 and the phosphorylation state of the inhibitory subunit of troponin. The positive inotropic effect of 100 nM dulaglutide in HAP was attenuated by 100 nM exendin (9–39). In contrast, dulaglutide alone or in the presence of phosphodiesterase inhibitor rolipram up to 100 nM failed to exert a positive inotropic effect in isolated electrically paced (1 Hz) mouse left atrial preparations and did not increase the beating rate of spontaneously beating mouse right atrial preparations. Our data suggest that dulaglutide raised force of contraction in the isolated paced human atrium via GLP-1R involving cAMP-dependent protein kinase in HAP.

## Introduction

**Fig. 1:**
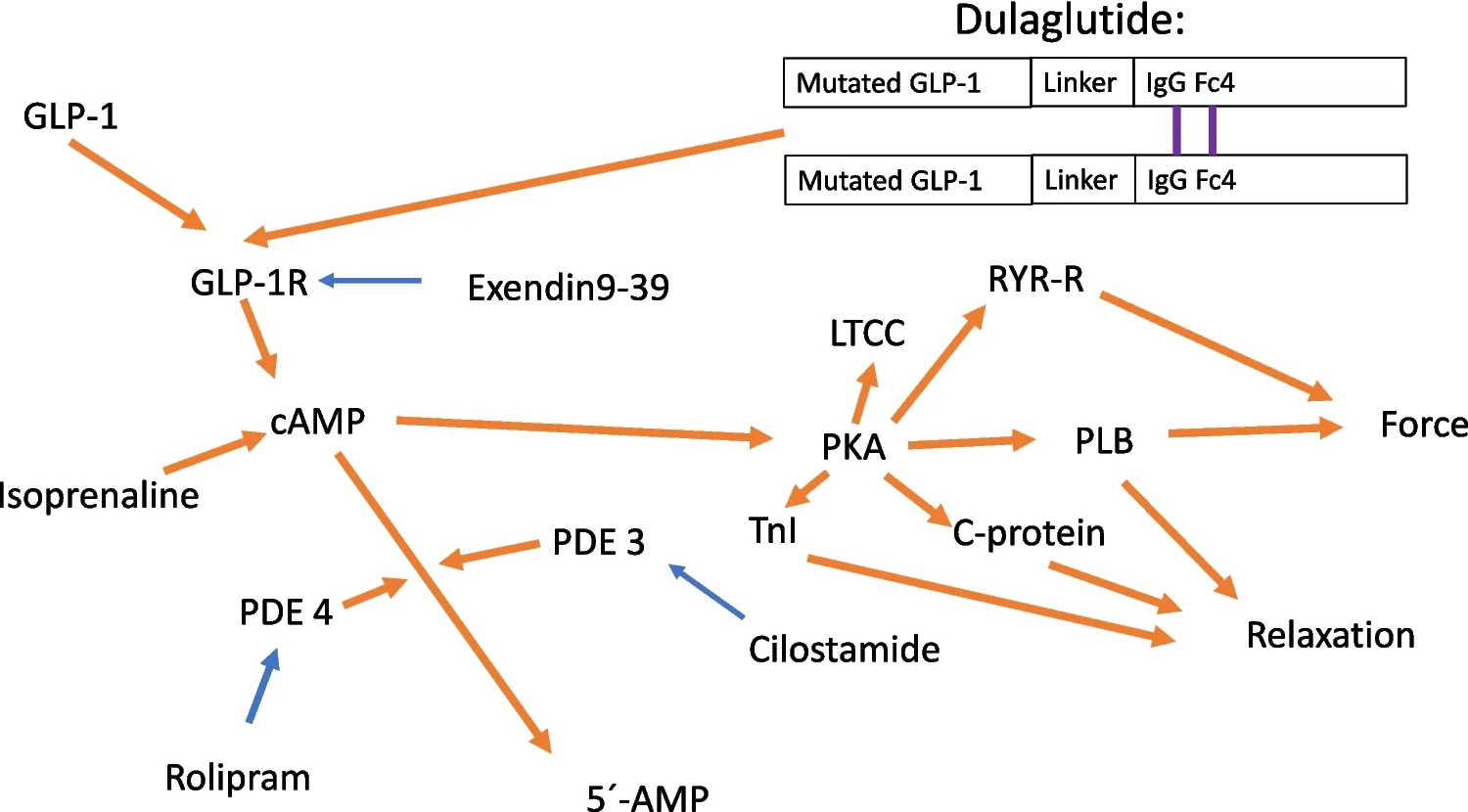
Putative mechanism(s) of action of dulaglutide in human cardiomyocytes. Endogenous GLP-1 stimulates (red arrow) the GLP-1 receptor (GLP-1R) to generate cAMP. The GLP-1R is antagonized (blue arrow) by exendin9-39. Isoprenaline also elevates cAMP. The cAMP then activates cAMP-dependent protein kinases (PKA). PKA phosphorylates and activates phospholamban (PLB), the ryanodine receptor (RYR, through which Ca^2+^ can leave the sarcoplasmic reticulum and thus increase force more rapidly and to a higher extent), the L-type calcium ion channels (LTCC, facilitating Ca^2+^ entry through the sarcolemma, leading to more generated force), the inhibitory subunit of troponin (TnI) and C-protein, both located in myofilaments. PLB can increase force of contraction in the heart and the muscle relaxation. RYR can also be phosphorylated by the Ca^2+^ -calmodulin dependent protein kinase (not shown). The cAMP is degraded by phosphodiesterases (PDE) to inactive 5′-AMP. PDE 3 and PDE 4 are inhibited by cilostamide and rolipram, respectively. Dulaglutide can be regarded as two identical peptide chains connected by disulfide bonds (pink arrows). The N-terminal part of dulaglutide starts with a mutated GLP-1 peptide which is connected via a linker to the mutated immunoglobulin G fragment abbreviated here as IgG Fc4

GLP-1 acts via GLP-1R (Fig. 1). GLP-1 augments in the pancreas the release of insulin. GLP-1 also inhibits in the pancreas the release of glucagon (Mayo et al. 2003). This reduces the blood glucose level in patients with type 2 diabetes (Drucker 2022). GLP-1 has a half-life of minutes. Hence, exogenous compounds with longer half-life than endogenous GLP-1, like dulaglutide, an approved drug in European Union and the USA, were developed to activate the GLP-1R in the pancreas (Drucker and Holst 2023). Dulaglutide like other GLP-1R agonists was beneficial for patients with regard to cardiovascular morbidity (Kristensen et al. 2019, Derington et al. 2025, Kwaah et al. 2025). Hence, there is a clinical interest in putative direct effects of dulaglutide on the human heart.

The GLP-1R (Fig. 1) like β-adrenoceptors couple via stimulatory guanosine triphosphate-binding proteins to adenylyl cyclases and raise intracellular cAMP levels in many cell types besides the pancreas (Mayo et al. 2003). Therefore, dulaglutide might augment also cardiac levels of cAMP, like the β-adrenoceptor agonist isoprenaline. If that were the case, dulaglutide should raise the force of contraction in HAP. Moreover, like isoprenaline, dulaglutide should, for instance, also increase protein phosphorylation of phospholamban, augment the rate of tension development, raise the rate relaxation should rise, shorten the time to peak tension and accelerate the time of relaxation. It was known when we started the present project that the GLP-1R agonists like GLP-1 or its more stable analogues exenatide or semaglutide or liraglutide elevate force of contraction in HAP (Wallner et al. 2015, Neumann et al. 2024).

Exendin(9–39), a proteolytic fragment of exenatide reduced the force of contraction stimulated by exenatide or GLP-1 in HAP (Serre et al. 1998, Wallner et al. 2015). From this is was concluded that the positive inotropic effect of exenatide in HAP is mediated by the GLP-1R (Fig. 1). Dulaglutide was developed in order to obtain a GLP-1R agonist that only needs to be given once a week (Glaesner et al. 2010). To that end, the GLP-1 sequence was mutated to slow degradation by human proteases (Glaesner et al. 2010). Moreover, the mutated core of a GLP-1 peptide was fused to a fragment of a human immunoglobulin also with the intention to prolonged the half-life of the compound and reduce immunogenic side effects (Fig. 1, Glaesner et al. 2010). Direct fusion of GLP-1 and the immunoglobulin fragment greatly diminished receptor affinity (Glaesner et al. 2010). Therefore, an array of small peptide linkers was tested. The best linker was used in dulaglutide and that linker prolonged the half-life but still did not impede the efficacy and potency of dulaglutide to raise cAMP in GLP-1R transfected cells (Glaesner et al. 2010). Nevertheless, the potency and the efficacy of dulaglutide to increase cAMP in cell culture was lower than that of exenatide (Glaesner et al. 2010). Hence, one can ask whether dulaglutide is effective enough at GLP-1R in the human heart to exert any positive inotropic effect. Moreover, mice are often use in preclinical diabetic research studies with dulaglutide (e.g. Deng et al. 2023, Jin et al. 2024; Xie et al. 2022). Hence, it might be helpful in this regard to know whether dulaglutide acts, at all, in the mouse heart and is thus an appropriate model for the human heart. Hence, one can ask whether dulaglutide like isoprenaline might increase the force of contraction in mouse left atrial preparations and beating rate in mouse right atrial preparations, respectively.

Hence, we tested the hypotheses that.

1. Dulaglutide raised force and protein phosphorylation in HAP.
2. Dulaglutide augmented force in mouse left atrial preparations and the beating rate in mouse right atrial preparations.

## Materials and methods

### Contractile studies in mice

The breeding, house-keeping and handling of the mice was in line with local animal protection requirements. In brief, the right or left atrial preparations from wild-type mice (CD-1, random sex, about 180 days old) were isolated and mounted in organ baths, as previously described (Neumann et al. 1998). The bathing solution of the organ baths contained a modified Tyrode´s solution: 119.8 mM NaCI, 5.4 mM KCI, 1.8 mM CaCl_2_, 1.05 mM MgCl_2_, 0.42 mM NaH_2_PO_4_, 22.6 mM NaHCO_3_, 0.05 mM Na_2_EDTA, 0.28 mM ascorbic acid and 5.05 mM glucose. The solution was continuously gassed with 95% O_2_ and 5% CO_2_ and maintained at 37 °C and pH 7.4 (Neumann et al. 1998, 2019). Spontaneously beating right atrial preparations from mice were used to study chronotropic effects and inotropic effects. Electrically stimulated (1 Hz) left atrial preparations from mice were used to study inotropic effects. We used this stimulatory rate for comparison with our previous studies (starting chronologically with Neumann et al. 1998). Moreover 1 Hz, we also used for stimulation of HAP, hence identical stimulation conditions were used in mice and man, probably facilitating comparison across species. Force was quantified in electrically paced isolated atrial preparations under isometric conditions. Duration of electrical stimulation with a rectangular impulse of direct current lasted for 5 ms. The voltage was 10% higher than necessary to initiate contraction. Muscles were stretched such that the maximum basal force was generated and then allowed to stabilise for 30 min before drug application started. Spontaneously beating right atrial preparations from mice were also used to study any chronotropic effects. The same basal beating rate as reported here, we have noted in our previous studies on mice (e.g. Neumann et al. 2024). Several different protocols for drug application were used as follows. After equilibration was reached, dulaglutide alone of first rolipram and then dulaglutide were added to LA or RA. Thereafter, isoprenaline was added in some cases as positive control (see legends to figures for details).

### Contractile studies on human preparations

Contractile studies on HAP were performed with the same setup and buffer used in the mouse studies above and have published these conditions repeatedly (e.g. Neumann et al. 2019, 2021). In brief, force of contraction was quantified in electrically paced (1 Hz) isolated left atrial preparations. We stimulated with because this is the physiological spontaneous beating rate in living humans. Duration of electrical stimulation with a rectangular impulse of direct current lasted for 5 ms. The voltage was 10% higher than necessary to initiate contraction. Muscles were stretched such that the maximum basal force was generated and then allowed to stabilise for 30 min before drug application started. Basal developed force can be seen in the relevant diagrams in this paper labelled with milli Newton (mN) in the ordinates under the condition labelled control (Ctr). The characteristics of patients are summarized in Table 1. The patients suffered from coronary diseases (two and three vessel diseases) or valve disease as main cardiac morbidities. Cardiac specific drug therapy included acetylsalicylic acid, anticoagulant drugs, loop diuretic drugs, β-adrenoceptor antagonists, and statins. Additional drugs in this patients were (in alphabetical order = amlodipine, atorvastatin, budesonide, candesartan, simvastatin, dapaglifloxin, empaglifoxin, ezetimibe, insulin, lamotrigine, metformin ramipril, valsartan, pantoprazol, pregabalin, oral iron preparations, sacubitril, sitagliptin, spironolactone and thiamazole. No patient received a GLP-1R agonist. Five patients suffered from type 2 diabetes. Cardiac comorbidities included in addition to angina pectoris also hypertension and atrial fibrillation. Additional morbidities that our patients presented were: alcoholism, anaemia due to iron deficiency, epilepsy, gastritis, hyperthyreosis, hyperlipidaemia, hyperuricemia, obesity, prostatomegaly, renal insufficiency and shingles.

**Table 1:** Clinical data for the patients studied. The cardiac relevant comorbidities and the cardiac related medications are indicated

Variables	N = 13 patients
Age (mean ± SEM)	73 ± 1.8
Male sex	10
Left ventricular ejection fraction (mean ± SEM)	49 ± 3%
Hypertension	8
Atrial fibrillation	3
Diabetes	5
Coronary artery bypass surgery	11
Valve operation	2
β-adrenoceptor antagonists	9
Angiotensin II receptor antagonists	3
Angiotensin converting enzyme inhibitor	7
Angiotensin receptor-neprilysin inhibitors	1
Sodium/glucose cotransporter 2 inhibitors	4
Mineralcorticosteroid receptor antagonists	2
Diuretics	6
GLP-1R agonists	0

In contraction experiments, dulaglutide was given cumulatively or as 100 nM and thereafter, in same cases, exendin(9–39) (Serre et al. 1998) was given. The drug application is depicted in the figures. Informed written consent was obtained from all patients included in the study. In the present work, we did not take into account a run down of the human muscle samples. Indeed, we have often shown that under our experimental conditions a run down of around 10% over one hour would occur (e.g. Neumann et al. 2024). However, we deliberately chose not to correct our data with run down controls because in this way we would underestimate but not overestimate the positive inotropic effect of dulaglutide. For comparison with our previous publications on human atrial samples (e.g. Neumann et al. 2024), we present here data in mN for the force of in percentage of pre-drug (before dulaglutide) value. This is done because we added after equilibration drugs to all HAP. Hence, each muscle is in this design of the experiments serves as its own control.

#### Western blotting

When the contraction studies in human atrial preparations (see above) had the intended inotropic effects, the sample was rapidly removed from the organ bath, put into a precooled plastic test tube (1.5 ml volume) usually in less than 10 s. The test tube was immersed in a beaker filled with liquid nitrogen. In this way all enzymatic processes were terminated. The homogenization of the frozen samples, protein measurements, electrophoresis, primary and secondary antibody incubation and quantification were performed following our previously published protocols (Gergs et al. 2010). In brief, the frozen human atrial preparations were mechanically homogenized in a frozen SDS/bicarbonate solution to inhibit any enzymatic processes. Samples were then allowed to thaw and protein content was determined in each sample. In each lane of a 20 lanes gel, 20 µg protein of these homogenates were loaded. Here, we performed electrophoresis using pre-cast gels (Novex™ 4–20% “Tris–Glycine Plus Midi Protein Gels”, Invitrogen, Thermo Fisher Scientific, Waltham, Massachusetts, USA). Thereafter, proteins were electrically transferred in a phosphate buffer to nitrocellulose membranes. Protein lanes were reversibly stained with Ponceau red and cut according to molecular weight horizontally using pre-stained molecular weight standards. Upper parts of the nitrocellulose membranes we incubated with an anti-calsequestrin antibody (#ab3516, abcam, Cambridge, England) and the remainder of the nitrocellulose membrane was incubated with an antibody against phospholamban phosphorylated at serine 16 or against total phospholamban (#A010-12 AP, A1, Badrilla, Leeds, England) or agonist phosphorylated TnI, as reported (e.g. Gergs et al. 2009). Thereafter, secondary horseradish peroxidase-coupled antibodies were added and signals were detected with an imager and quantified with densitometry (Amersham ImageQuant 800, Cytiva, Freiburg im Breisgau, Germany). The ratio of serine 16-phosphorylated phospholamban and calsequestrin was calculated and is reported. We have further confirmed before that the antibody we are using identifies phospholamban in human atrial homogenates using boiling. This heat treatment in the SDS containing sample buffer interrupts the pentameric interaction of phospholamban monomers. This removes the high molecular band recognized by the antibody we used (Wegener and Jones 1984; Gergs et al. 2009). Moreover, we have identified before troponin by using radioactivity labelled myocytes and incubating the Western blot with our antibody (Neumann et al. 1994). Calsequestrin was identified by using this antibody in wild-type mouse hearts and hearts of calsequestrin 2 knock-out mice we had ourselves generated. In the lanes from the calsequestrin 2 knock-out mice the signal had vanished (Gergs et al. 2017).

### Data analysis

Data shown are the means ± standard error of the mean. Statistical significance was estimated using the analysis of variance (ANOVA) followed by Bonferroni’s *t*-test or paired two-tailed Student’s *t*-test, as appropriate. A *p*-value < 0.05 was regarded as significant. Prism® software was used for statistical analysis and visualisation of data.

### Drugs and materials

Dulaglutide came from MedChemExpress (local distributor: Hycultec, Beutelsbach, Germany); exendin(9–39) was from Thermoscientific (local distributor: Th. Geyer, Renningen, Germany), and exendin (9–39) was supplied by Toronto Research (local distributor: Biozol, Eching, Germany). All other chemicals were of the highest purity grade commercially available. Deionised water was used throughout the experiments to prepare the modified Tyrode’s solution. Stock solutions were prepared daily.

## Results

**Fig. 2:**
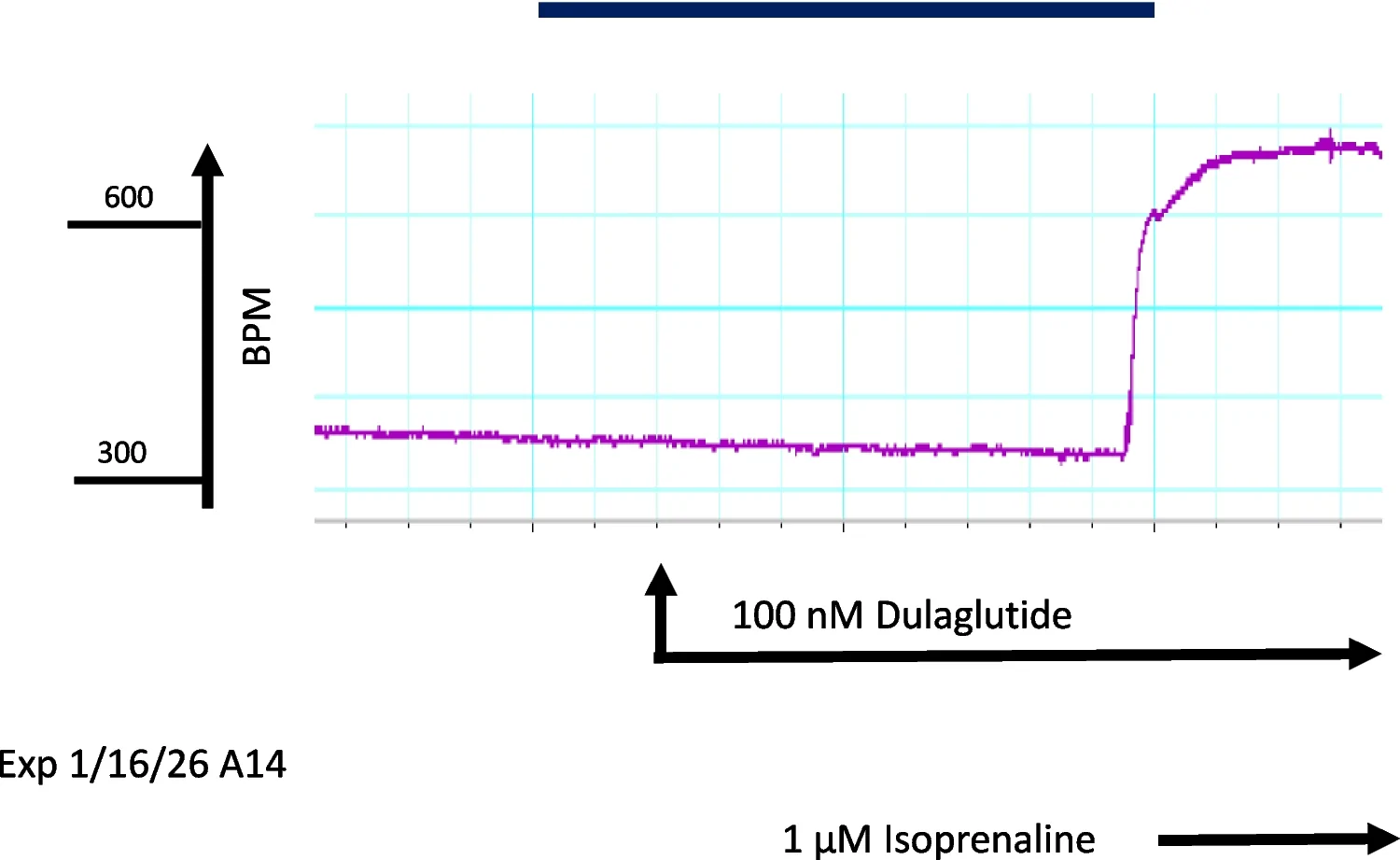

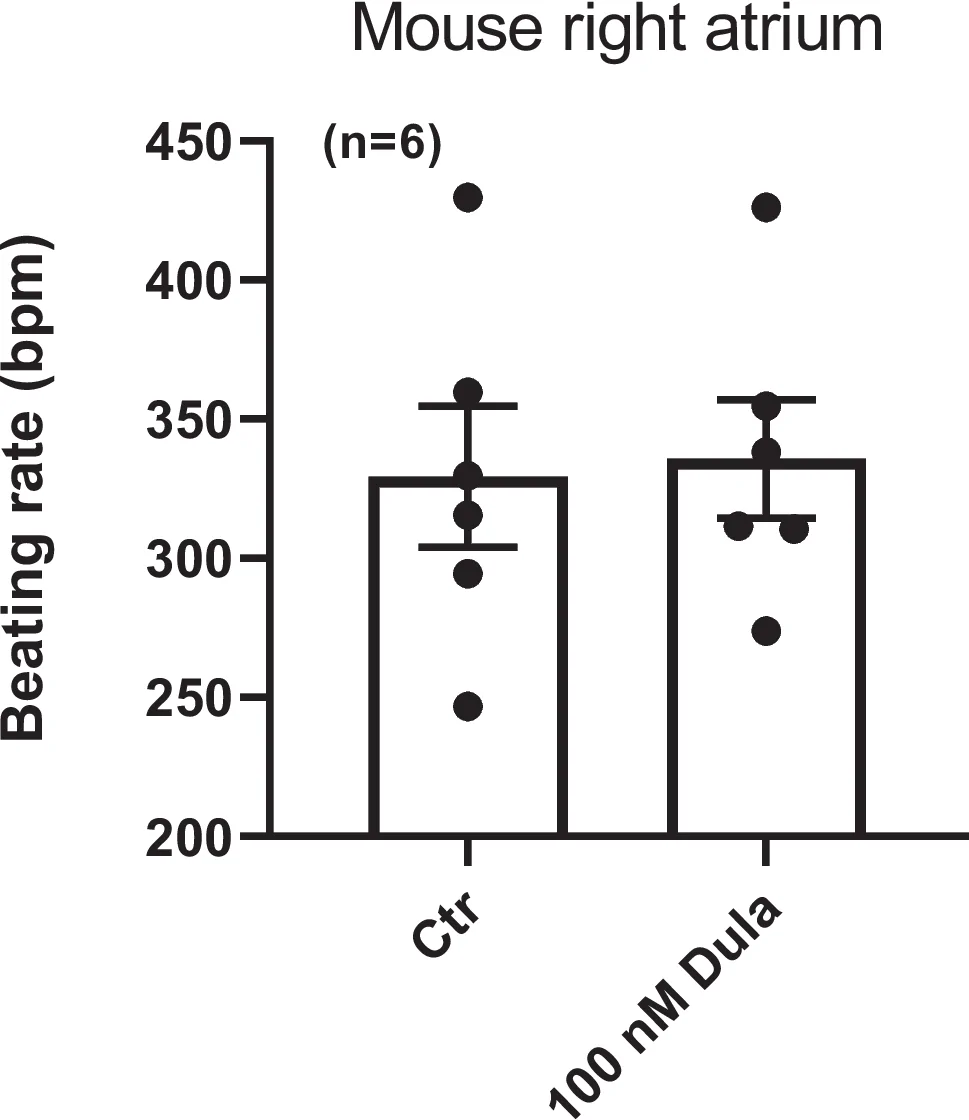

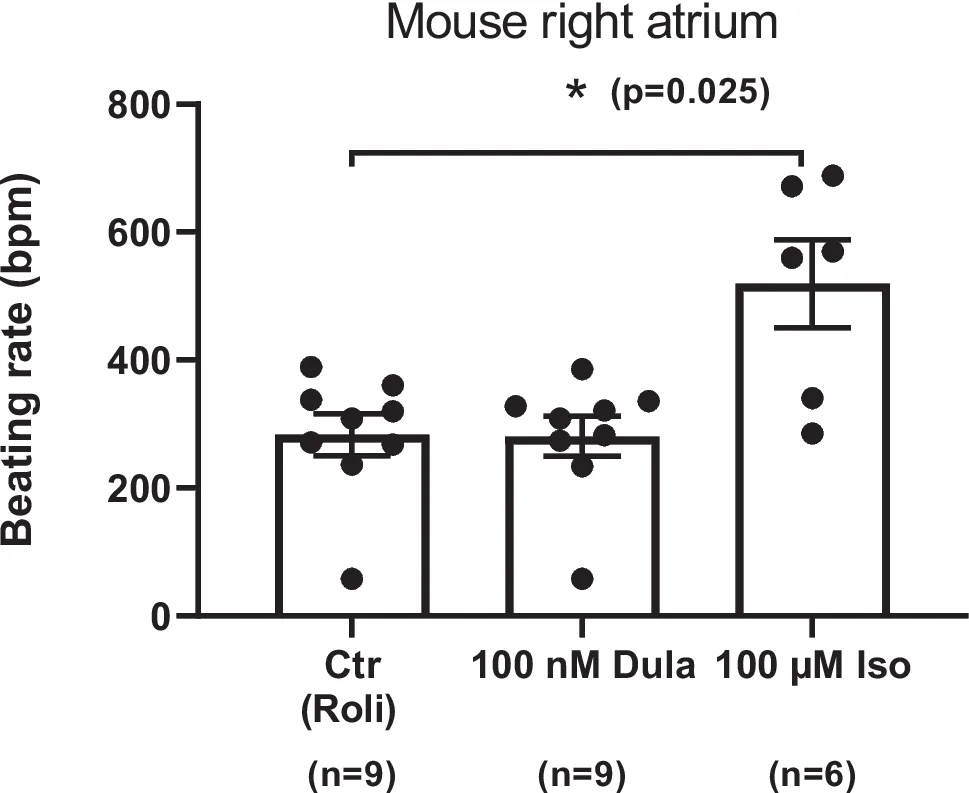
Dulaglutide failed to increase the beating rate in mouse atria. (**A**) Original recoding of beating rate of 100 nM dulaglutide alone and then 1 µM isoprenaline in mouse right atrial preparations. (**B**) Bar diagram of the effects of 100 nM dulaglutide (Dula) alone on beating rate in isolated mouse right atrial preparation. (**C**) Effects of dulaglutide 100 nM (Dula) or 1 µM isoprenaline (Iso) in the presence of 100 nM rolipram(Roli) on the beating rate in right atrial preparations. Ordinates in the Figure indicate beats per minute (bpm). Numbers in brackets indicate number of experiments. * indicates significant differences (compare *p*-values next to asterisks) versus Ctr (pre-dulaglutide values) or after 100 nM rolipram (Roli)

For comparative purposes, we studied initially mouse right atria and then left atria. A putative cAMP-increasing agent such a dulaglutide is expected to induce tachycardia. Therefore, we questioned whether dulaglutide would raise the beating rate in right atrial preparations. We first interrogated the effect of dulaglutide in the absence and then in the presence of rolipram, a phosphodiesterase 4 inhibitor, on the beating rate in mouse right atrial preparations. However, in the absence of rolipram, dulaglutide failed to increase the beating rate. This is depicted in an original recording in Fig. 2A. Dulaglutide was unable to increase or decrease the beating in RA, whereas 1 µM isoprenaline, used a positive control increased the beating rate (Fig. 2A) suggesting that the right atrial preparation can respond with a tachycardia to cAMP-increasing agents, at least in principle. Several experiments are summarized in Fig. 2B. The data show that dulaglutide (Dula) alone did not increase the beating rate compared to pre-drug values (Ctr, Fig. 2B). In further experiments, we initially added rolipram (100 nM) to the organ bath. After 10 min we added 100 nM dulaglutide and thereafter, we further added 1 µM isoprenaline. Again isoprenaline was utilized at a positive control. We summarized several experiments in Fig. 2C. Under these conditions 100 nM dulaglutide failed to raise beating rate above values obtained with rolipram (Roli). Only isoprenaline (Iso) significantly increased beating rate under these experimental conditions (Fig. 2C).

**Fig. 3:**
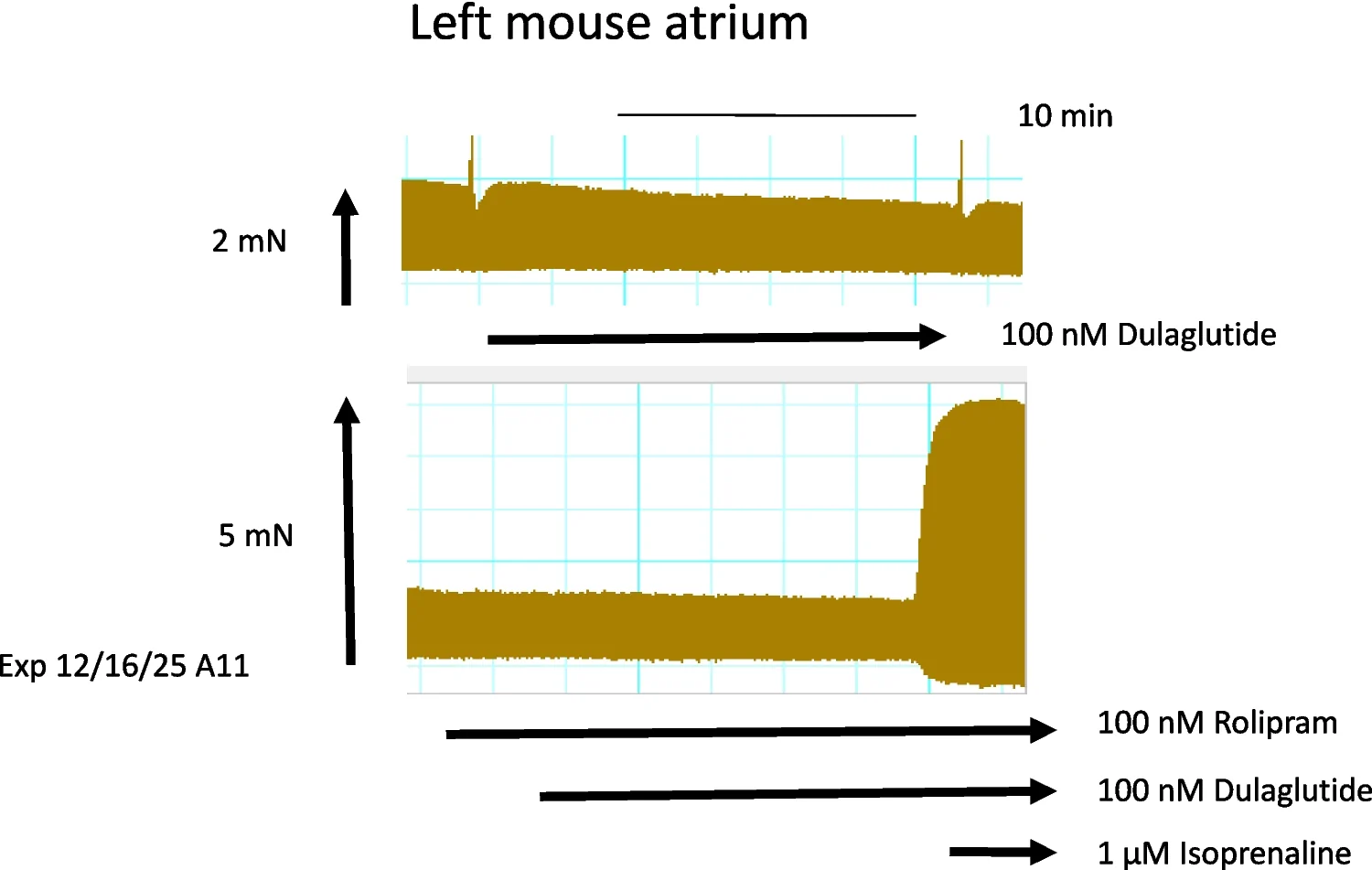

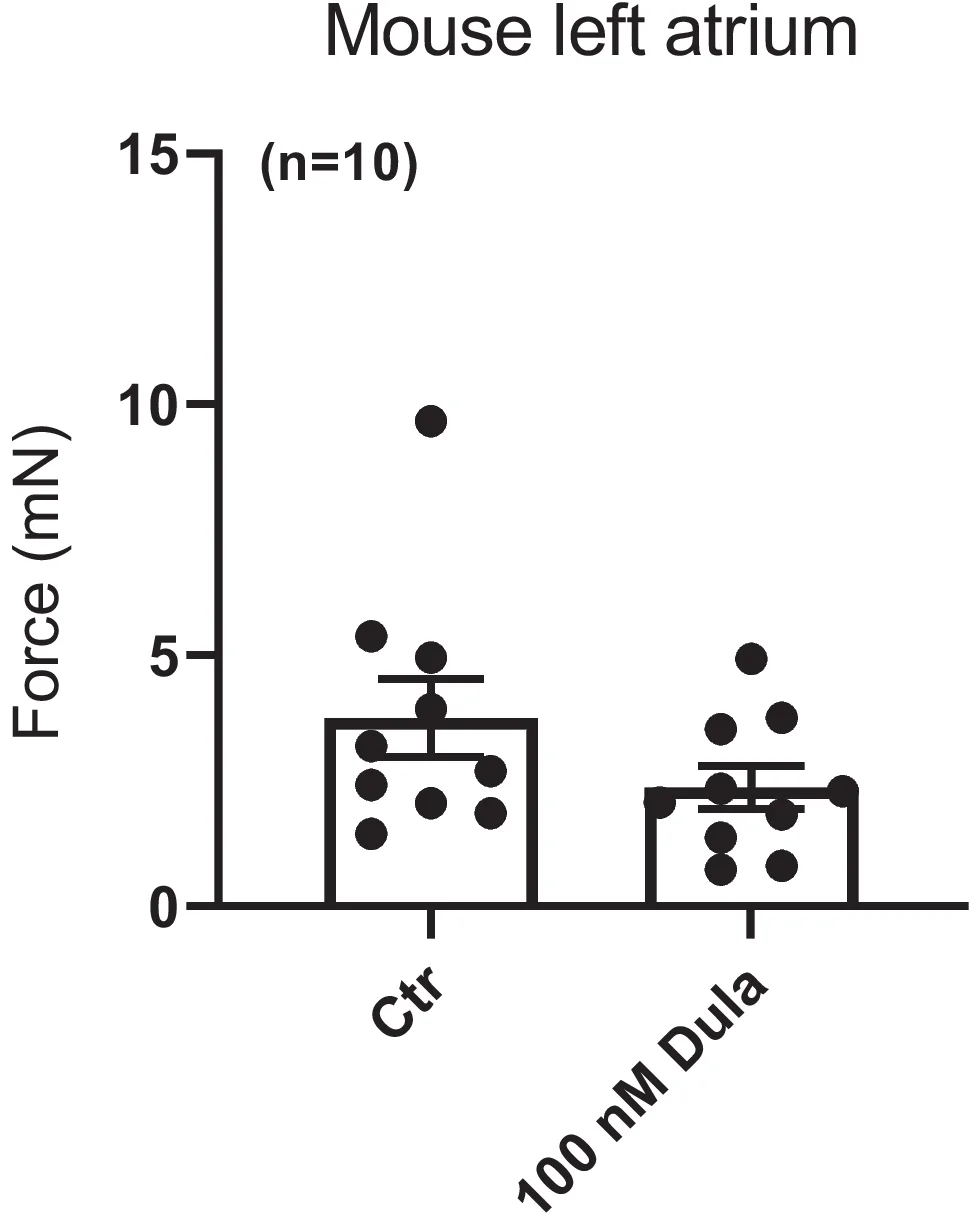

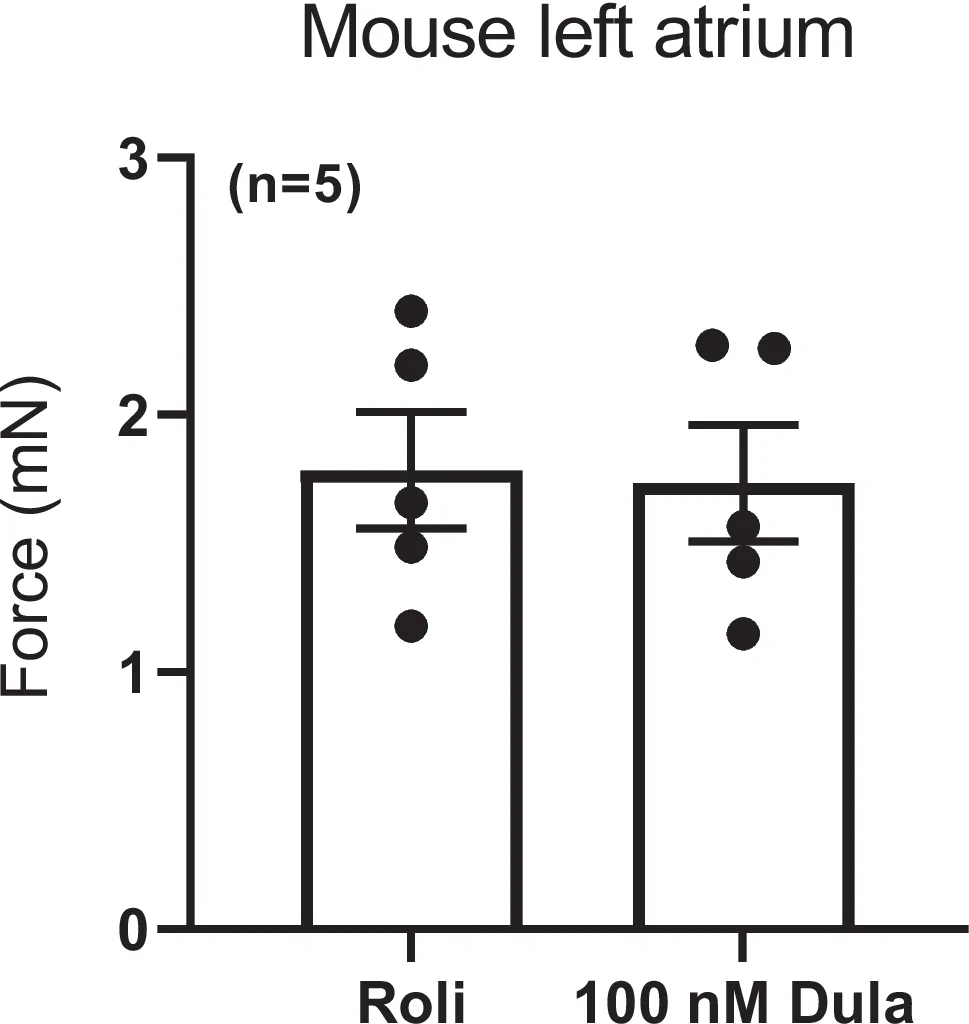
Dulaglutide failed to increase the force of contraction in mouse atria. Original recordings in wild type mouse left atrial preparations. Effect of dulaglutide 100 nM alone (**A**, top) or in the presence of 100 nM rolipram and then 1 µM isoprenaline (**A**, bottom) on force of contraction in isolated left mouse atrial preparations. Bar diagram on the effects of 100 nM dulaglutide (Dula) versus pre-drig value (Ctr) on force of contraction (**B**). Bar diagram on the effects of 100 nM dulaglutide (Dula) in the presence of 100 nM rolipram versus pre-drug value (Roli) on force of contraction (**B**). Arrows in (**A**) and ordinates in (**B**) and (**C**) give force of contraction in milliNewton (mN). Horizontal bar indicates time in minutes (min) (**A**, top). Numbers in brackets indicate the number of experiments. * indicate significant differences versus Ctr or Roli

A putative cAMP-increasing agent such a dulaglutide was expected to raise force of contraction in mouse left atrial preparations. Hence, we examined whether dulaglutide could augment the force of contraction in mouse left atrial preparations. However, dulaglutide at 100 nM was unable to raise force of contraction in left atrial preparations. This result was depicted in an original tracing in Fig. 3A (top). Several such experiments on force of contraction were summed up in Fig. 3B. However, dulatglutide failed to raise force of contraction above pre-drug levels (Ctr). The effects of cAMP-increasing agents on force of contraction can often be augmented by a phosphodiesterase inhibitor. Hence, we repeated the experiment with dulaglutide in the presence of 100 nM rolipram, a drug we used repeatedly in mice at this concentration to potentiate cAMP-mediated effects on force of contraction in mouse atrial preparations (e.g. Neumann et al. 2019). However, also in the presence of rolipram, 100 nM dulaglutide did not elicit an augmentation in force of contraction in left atrial preparations of mice. This is seen in an original preparation in Fig. 3A (bottom). In contrast, isoprenaline elevated force of contraction in left atrial preparations after rolipram from WT mice (Fig. 3A, bottom). Such data were summarized in Fig. 3C. There was no detectable positive inotropic effect of 100 nM dulaglutide compared to pre-treatment with 100 nM rolipram (Roli).

**Fig. 4:**
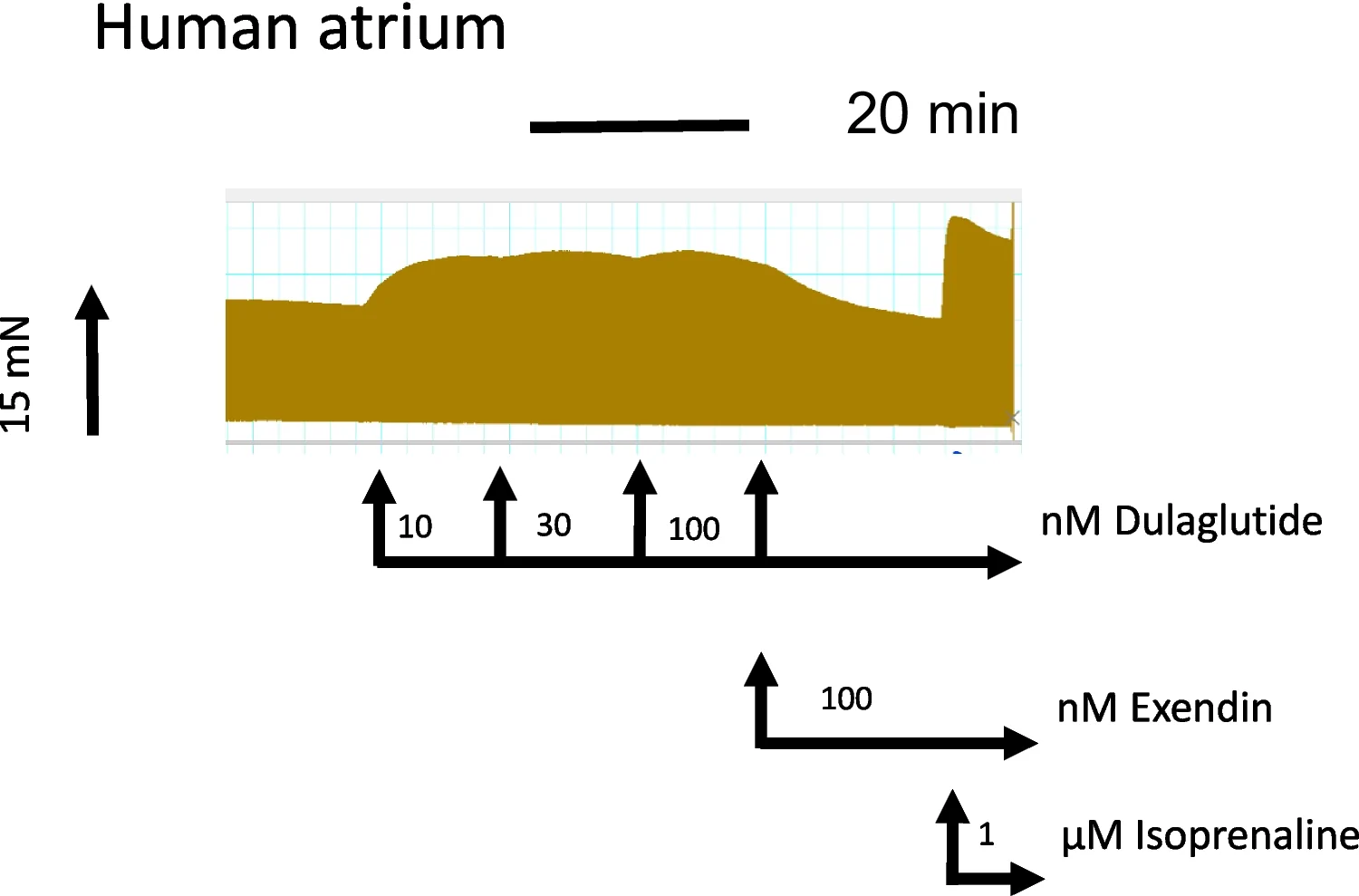

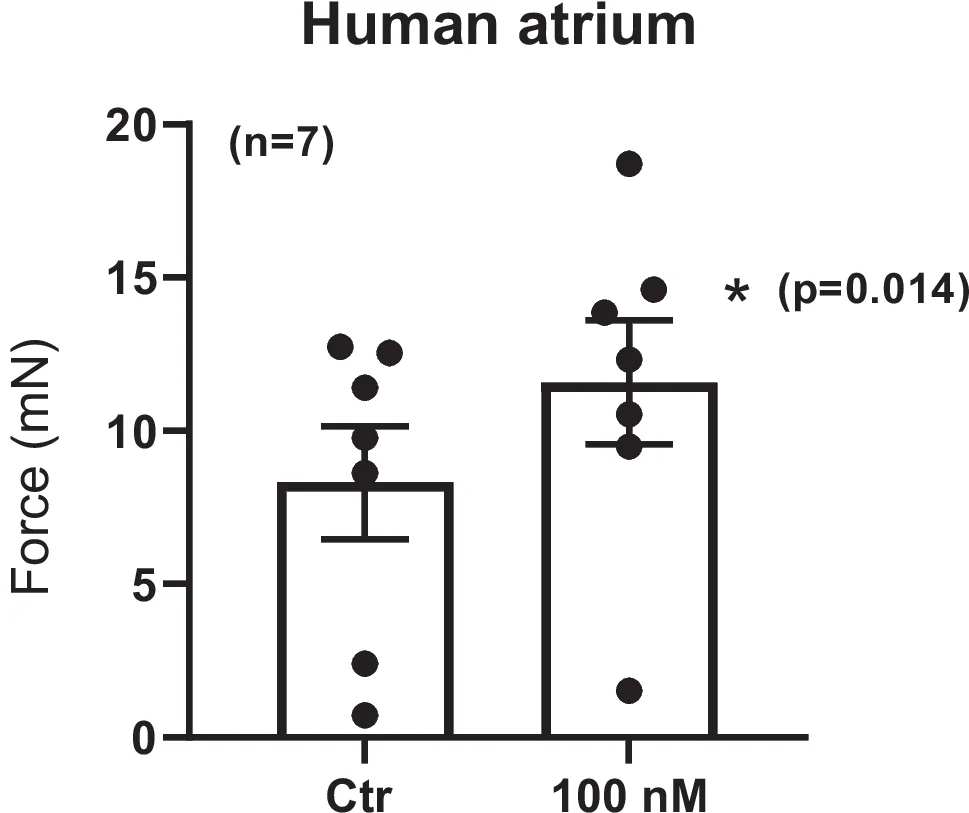

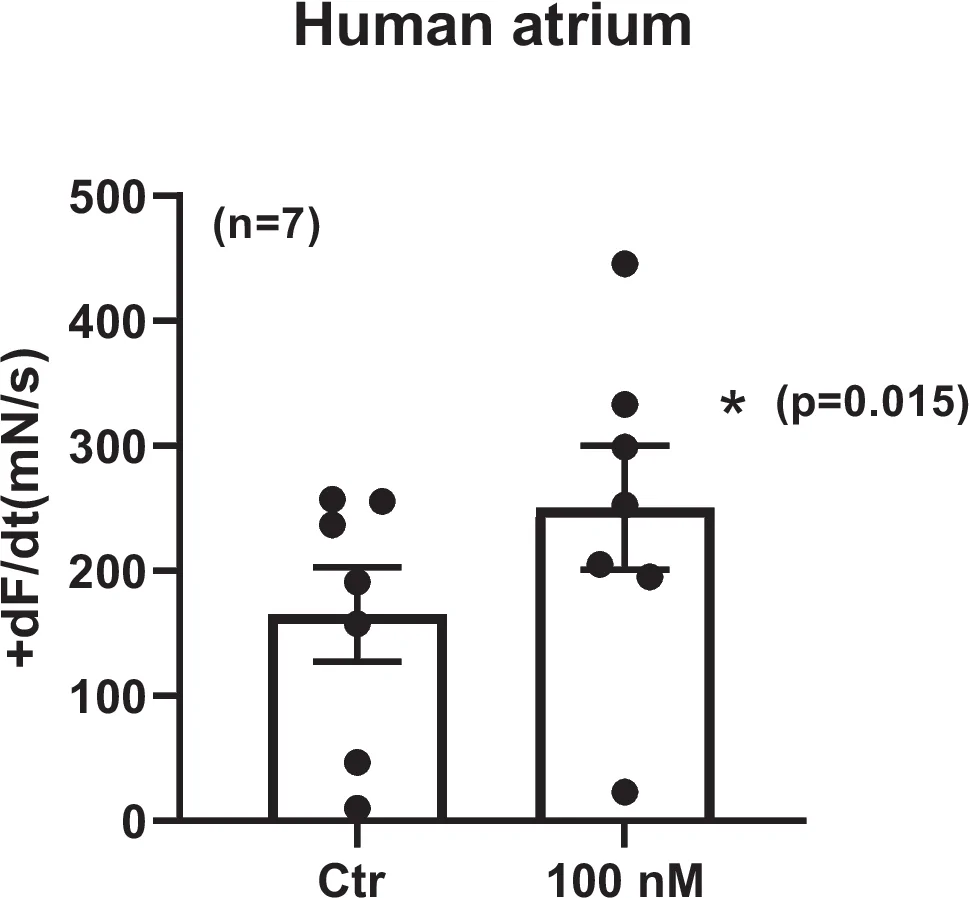

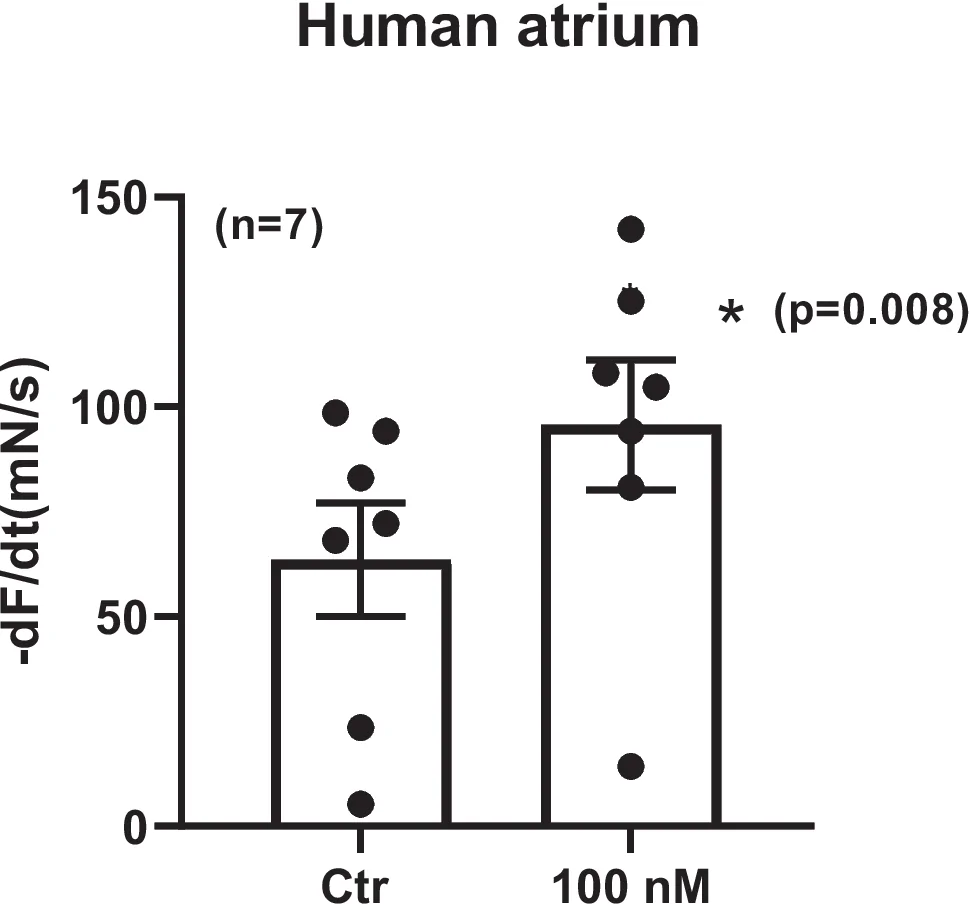

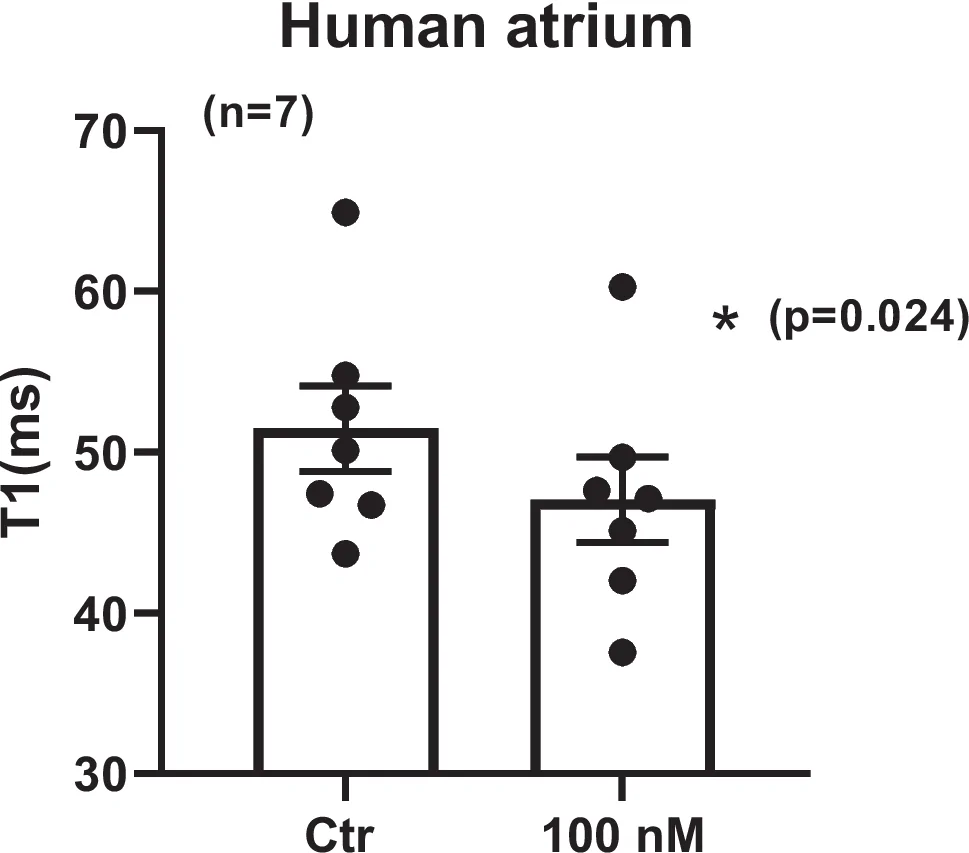

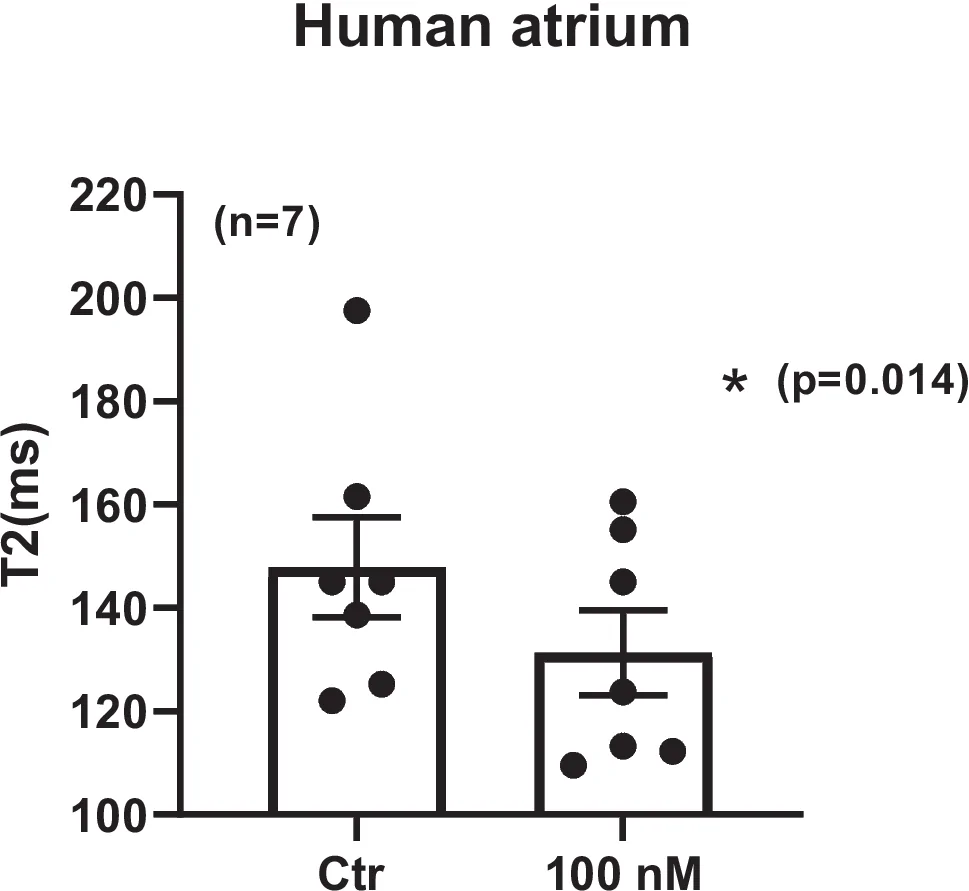
Dulaglutide alone increased force of contraction in HAP. Original recordings in HAP (**A**). Dulaglutide cumulatively applied (10–100 nM), induced a time-dependent and concentration-dependent positive inotropic effect in HAP (Fig. 4A). Subsequently, exendin9-39(exendin) reduced and a maximum effective concentration of isoprenaline (1 µM) increased force of contraction in HAP (**A**). Effects of 100 nM dulaglutide (Dula) compared to controls (Ctr) on force of contraction in mN are presented in (**B**). Moreover, the effect of 100 nM dulaglutide in HAP on the rate of tension development (**C**), on the rate of tension relaxation (**D**), time to peak tension (**E**) and time of tension relaxation (**F**) are plotted Ordinates indicate force in (**A**) and (**B**), or the maximum (+ dF/dt) or minimum (− dF/dt) rate of tension development in mN per second (mN/s) in (**C**) and (**C**) and in milliseconds (ms) in (**E**) and (**F**). * (next to the exact *p*-values in brackets) indicate significant differences versus Ctr (pre-dulaglutide values)

In contrast to left and right atrial preparations from from mice, we noted for dulaglutide a positive inotropic effect in HAP. The effect of dulaglutide given alone is seen in an original tracing in HAP (Fig. 4A). We detected a concentration-dependent and a time-dependent positive inotropic effect of dulaglutide in HAP (Fig. 4A). The effect seemed to start at 10 nM and was highest at 100 nM dulaglutide, the highest concentration we tested in this study. As the effects of 100 nM dulaglutide reached a maximum (Fig. 4A), we concentrated on 100 nM dulaglutide in the subsequent experiments in HAP. Such effects of 100 nM dulaglutide on force of contraction were summarized in Fig. 4B. It was apparent that 100 nM dulaglutide raise force of contraction in HAP compared to pre-drug value (Ctr). Moreover, dulaglutide (100 nM) brought about a significant rise in the maximum rate of tension development (Fig. 4C), the maximum rate of tension relaxation (Fig. 4D) and reduced the time to peak tension and time to relaxation (Fig. 4E).

**Fig. 5:**
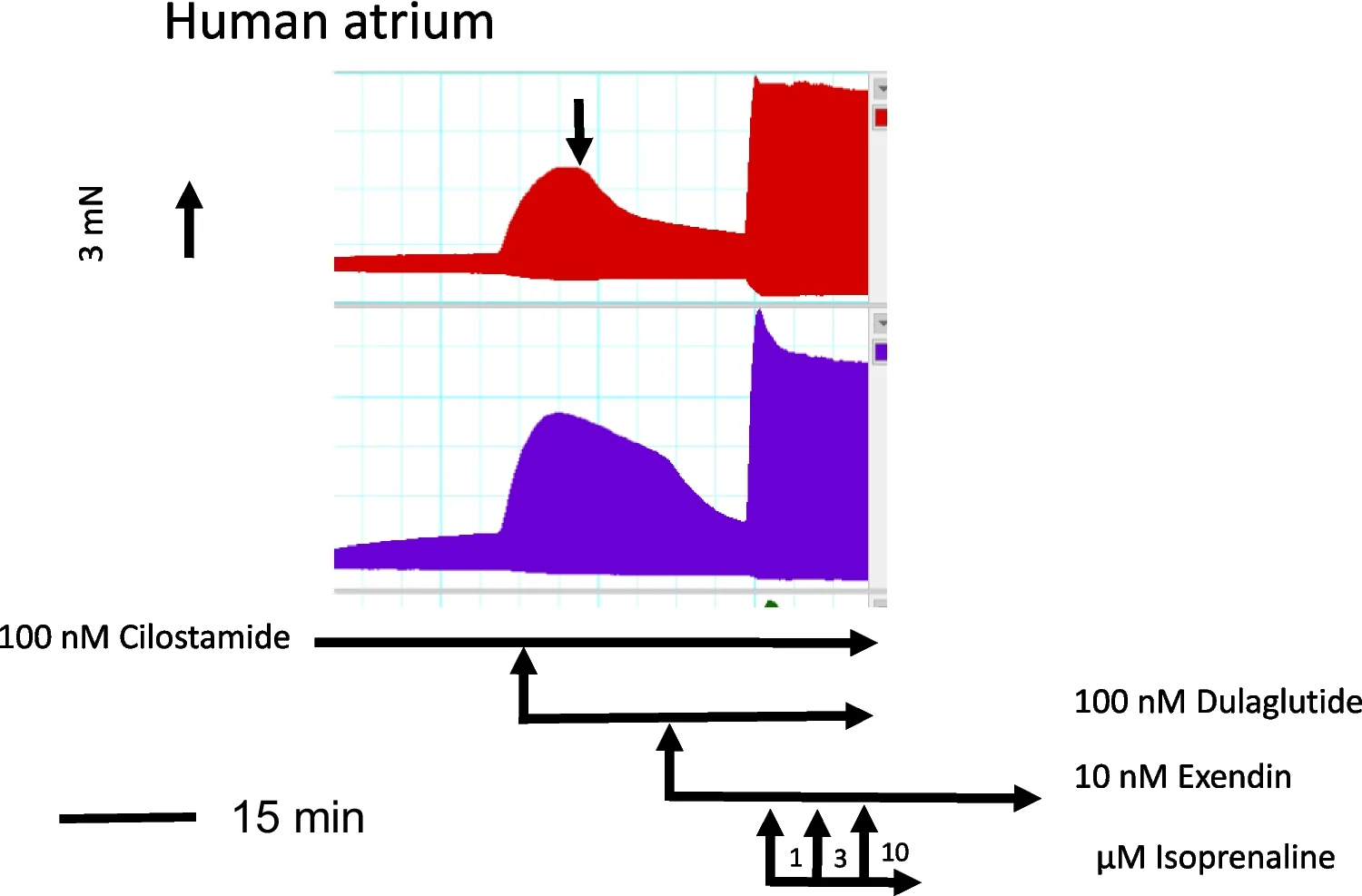

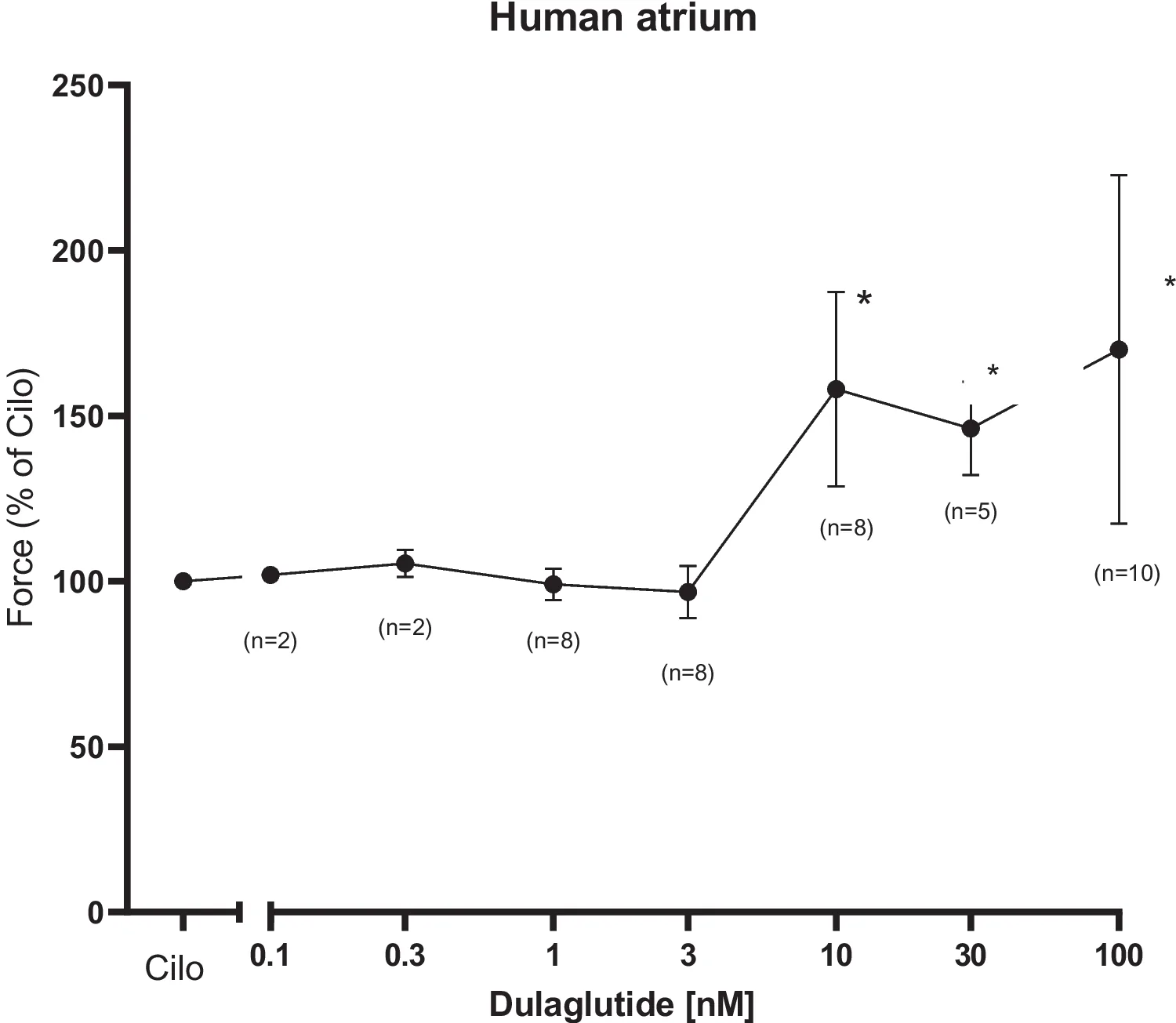

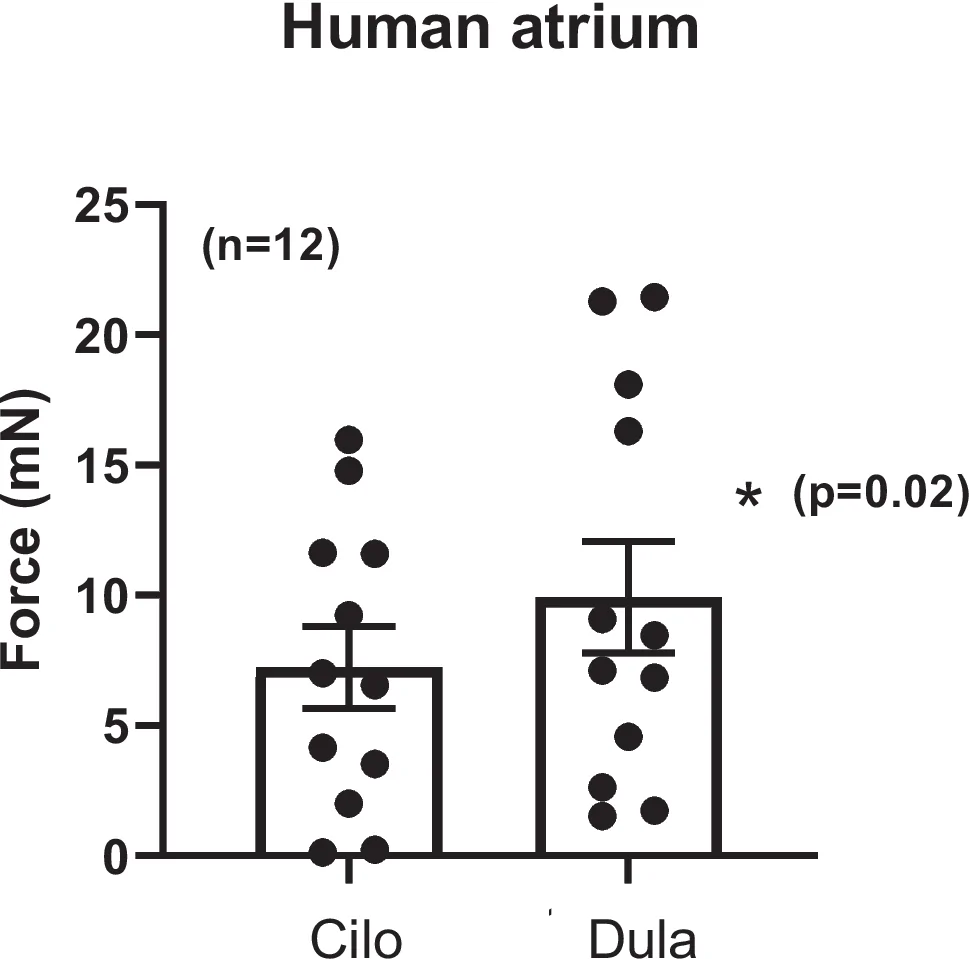

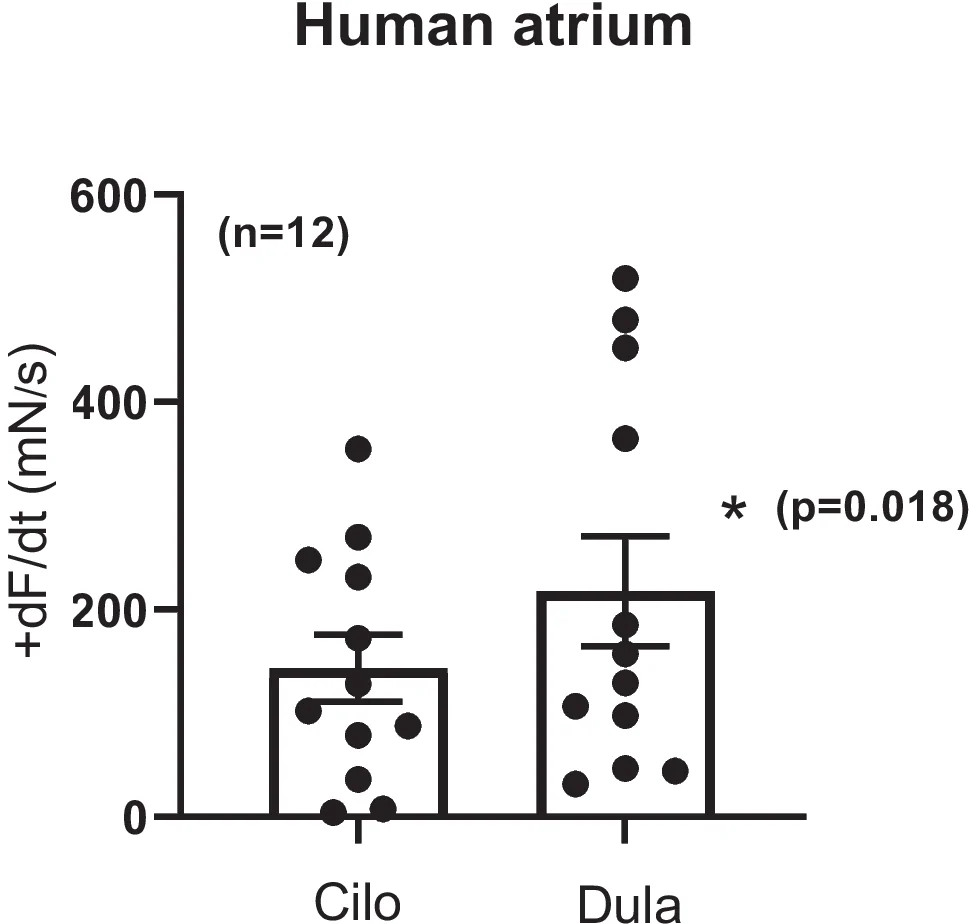

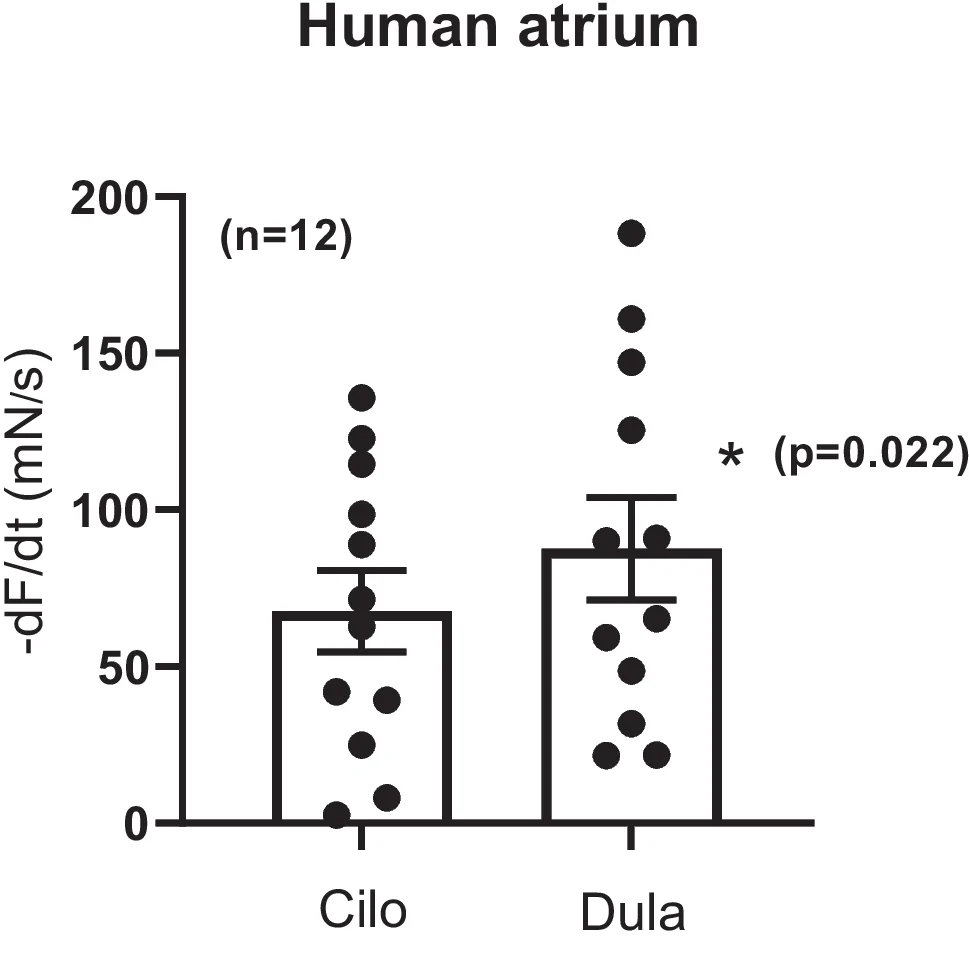

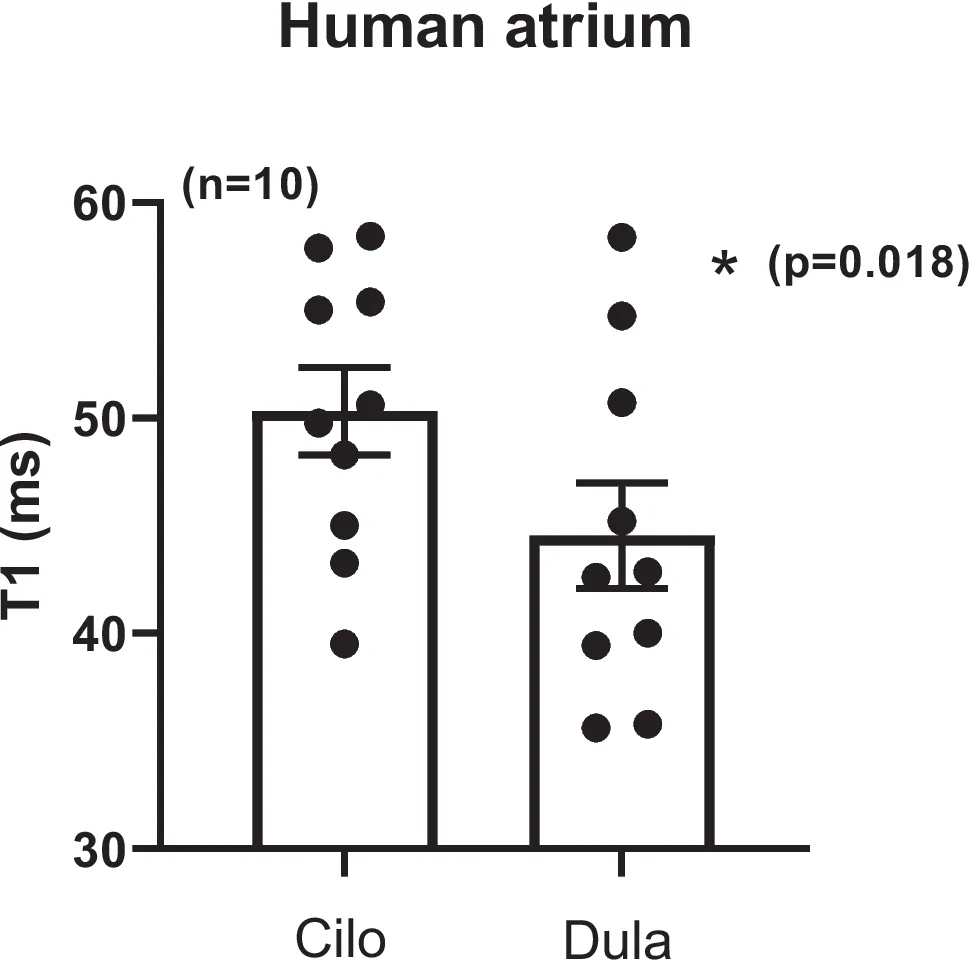

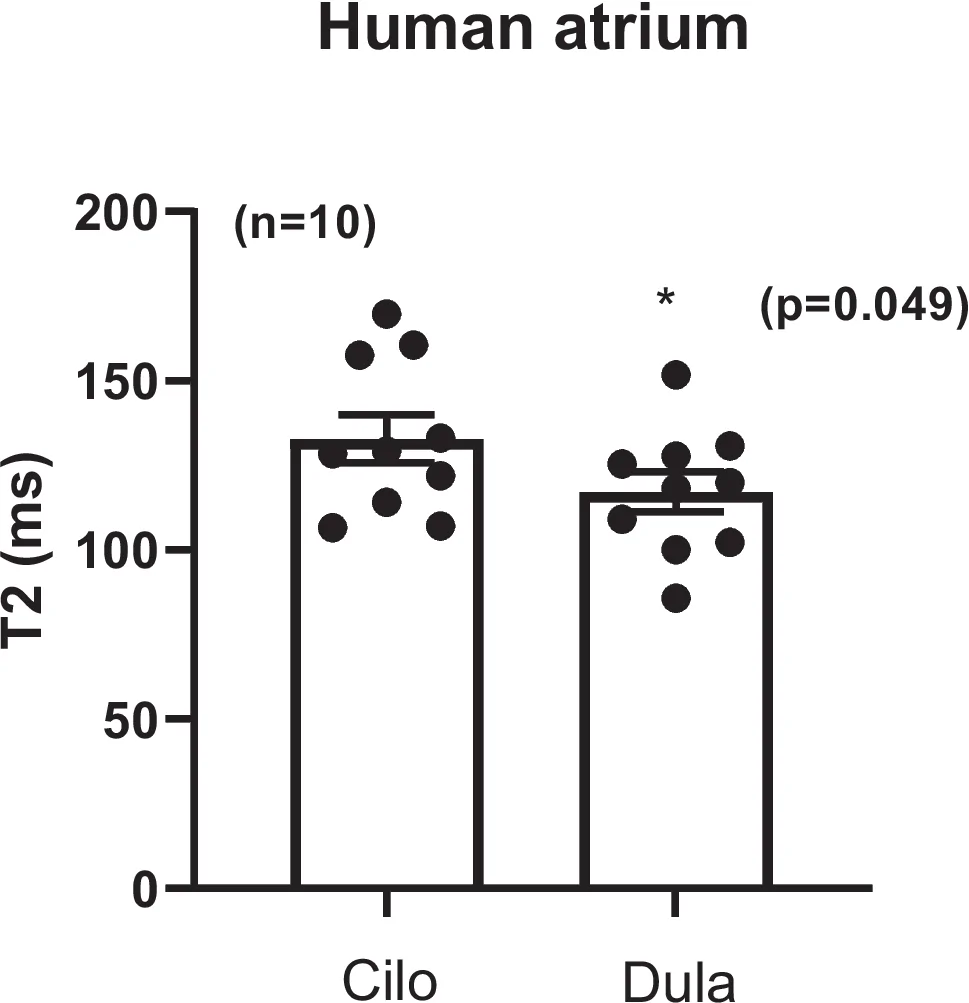
Dulaglutide in the presence of cilostamide increased force of contraction in HAP. Two different original recordings in HAP from one patient are depicted in (**A**). Dulaglutide in the presence of 100 nM cilostamide induced a time-dependent-dependent positive inotropic effect in HAP (**A**). Subsequently, exendin9-39(exendin, vertical arrow) reduced and isoprenaline (1 µM) again increased force of contraction. We display a concentration-response curve for the effect of dulaglutide in the presence of 100 nM cilostamide (Cilo) on force of contraction in % of the effect of Cilo ((**B**), Cilo = 7. 8 mN ± 0.85 mN). Moreover, we display effect of 100 nm Dulaglutide in the presence of 100 nM cilostamide on force of contraction ((**C**), in mN), on the maximum rate of tension development (dF/dt_max_, (**D**) in mN/s), on the rate of tension relaxation (dF/dt_min_, (**E**) in mN/s) on the time to peak tension ((**F**), in milliseconds, ms), and time to relaxation (**G**). Abscissa in (**B**) indicates concentrations of dulaglutide in negative decadic logarithm of molar concentrations. First significant differences versus 100 nM cilostamide (Cilo) are indicated in asterisk in (**B**). Otherwise asterisks (next to exact *p*-values in brackets) indicate significant differences between values for 100 nM dulaglutide (Dula) and 100 nM cilostamide. Integer numbers in brackets mean number of experiments

**Fig. 6:**
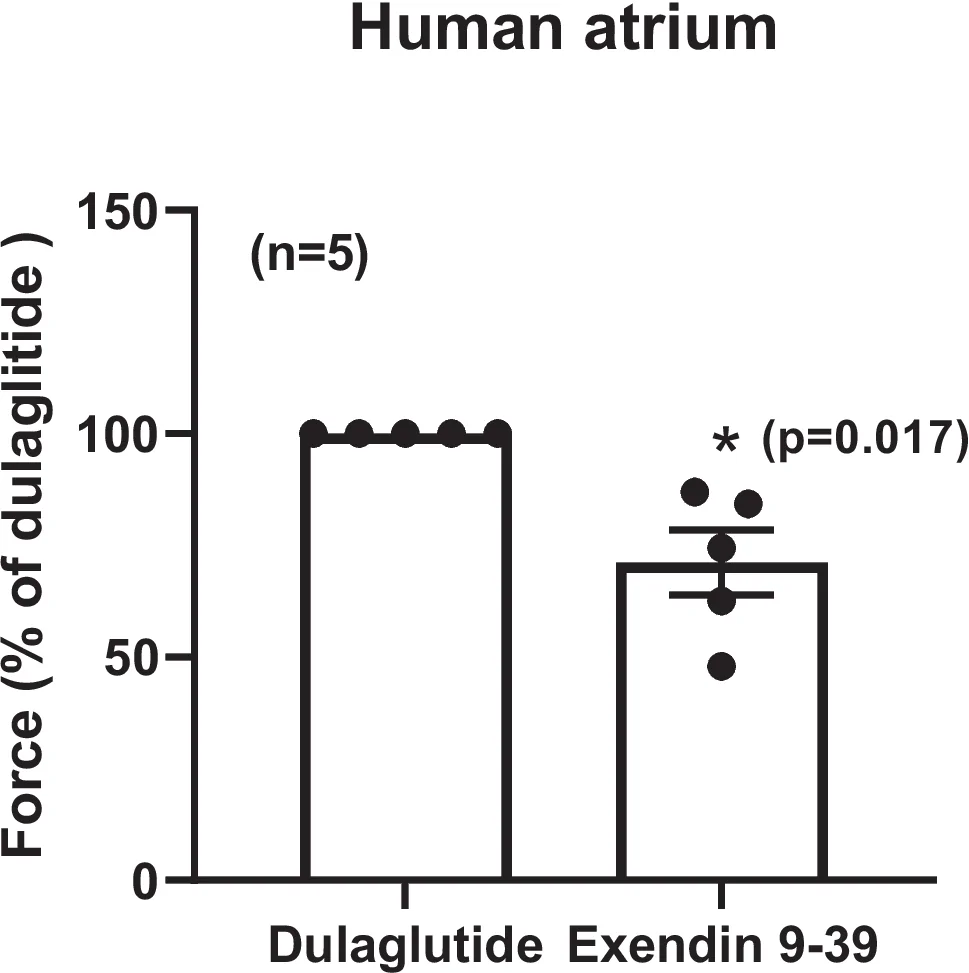
Dulaglutide acts via GLP-1R in HAP. Antagonism at GLP-1R. Dulaglutide (100 nM) raised force of contraction in the presence of 100 nM cilostamide in HAP to 8.9 ± 0.88 mN (left hand side bar). Subsequently applied 100 nM exendin9-39 reduced force of contraction (right hand side bar). Ordinate gives force of contraction in percentage of the effect of 100 nM dulaglutide before exendin9-39 was added in HAP. Number in brackets means number of experiments. * (next to the exact p-value) indicates a significant decrease versus the inotropic effect of 100 nM dulaglutide

Whereas the main phosphodiesterase in the mouse heart is phosphodiesterase 4, in the human heart the main phosphodiesterase is the phosphodiesterase 3 (Kamel et al. 2023). In the presence of a phosphodiesterase 3 inhibitor, namely 100 nM cilostamide, which we used before at this concentration in HAP (e.g. Neumann et al. 2024), dulaglutide led to a time-dependent positive inotropic effect in the HAP. This could be seen in two original tracings in Fig. 5A. Results from additional experiments are depicted in Fig. 5B. Here, complete cumulative concentration–response curves for dulaglutide on force of contraction in HAP were summarized versus pre-drug values, in this case 100 nM cilostamide (Cilo, Fig. 5B). We found that a significant positive inotropic effect started with 10 nM dulaglutide and reached its maximum at 100 nM dulaglutide. Therefore in further quantifications, we focused on the contractile effect of 100 nM dulaglutide. Indeed, in the presence of 100 nM cilostamide, dulaglutide (100 nM) heightened the force of contraction (Fig. 5C), the rate of tension development (Fig. 5D), the rate of tension relaxation (Fig. 5E) and reduced the time to peak tension (Fig. 5F) and time to relaxation (Fig. 5G). The positive inotropic effect of dulaglutide in the presence of cilostamide in HAP was reversed when we added the GLP1-R antagonist exendin(9–39). This can be seen in two original experiments in Fig. 5A. Such data are summarized in Fig. 6. It was apparent that exendin(9–39) reduced significantly force of contraction compared to the stimulation of the HAP with 100 nM Dulaglutide (Fig. 6).

**Fig. 7:**
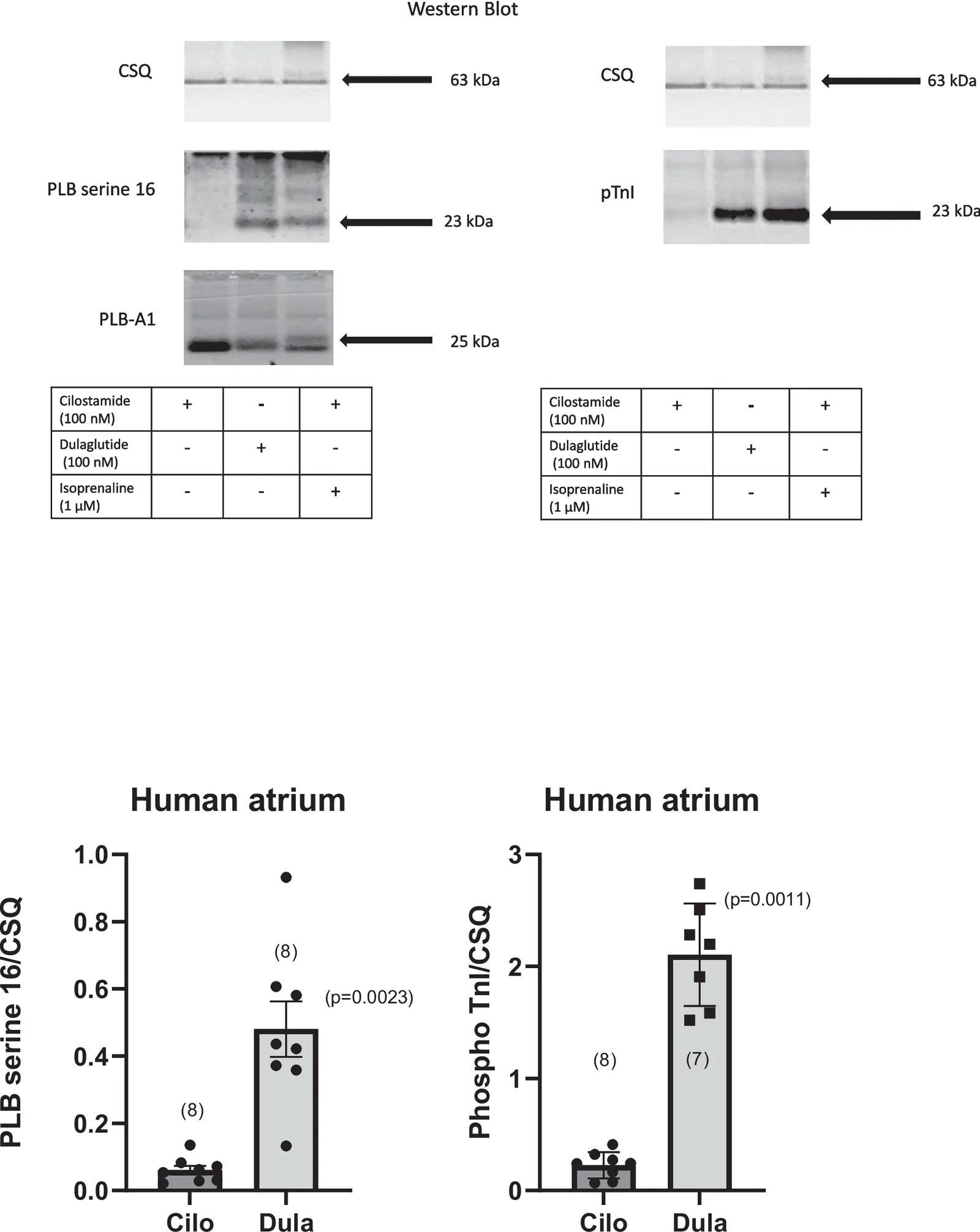
Dulaglutide increases the phosphorylation state of phospholamban in HAP. Both dulaglutide 100 nM (Dula) and 1 µM isoprenaline increased phosphorylation of regulatory proteins in the presence of 100 nM cilostamide (Cilo) in paced (1 Hz) isolated contracting strips from the human right atrium. Effects of 100 nM dulaglutide on phospholamban phosphorylation at serine 16 (PLB Ser16) is seen on the Western blot on the left hand side (**A**). The phosphorylation of troponin I (pTnI) is depicted on the Western blot on the right hand side (**B**). Arrows indicated PLB at about 23 kDa and TnI at about 31 kDa. As a loading control we assessed the protein expression of calsequestrin (CSQ) at about 63 kDa (arrow) by cutting the lanes of the blot and incubating the parts with different primary antibodies. Blots were quantified by densitometry to calculate the ratio signal for phospholamban phosphorylated at amino acid 16 and calsequestrin from the same lanes (**C**). Blots were also quantified by densitometry to calculate the ratio signal for phosphorylated troponin inhibitor (Phospho TnI) at amino acid 16 and calsequestrin (CSQ) from the same lanes (**D**). Numbers in brackets indicate the number of patients. Asterisks denote *p* < 0.05 using two-tailed unpaired Student’s *t*-test versus cilostamide alone (Cilo)

Finally, the question arose whether 100 nM dulaglutide in the presence of 100 nM cilostamide would alter the phosphorylation state of regulatory proteins. As a negative control, we treated the HAP with only 100 nM cilostamide. As a positive control, we treated the HAP with 100 nM cilostamide and added 1 µM isoprenaline. In line with the findings in Fig. 5, 100 nM dulaglutide in the presence of 100 nM cilostamide increased force of contraction. We rapidly freeze-clamped the contracting samples. In homogenates from these samples, we measured the phosphorylation state of phospholamban at serine 16 and the phosphorylation state of the inhibitory subunit of troponin with Western blotting using phosphorylation specific primary antibodies (Fig. 7A and B). These phosphorylations were referred to the expression of calsequestrin, a protein not regulated at the protein level by the cAMP-dependent protein kinase. Hence, we calculated the ratio of phosphorylation states of phospholamban and the troponin inhibitor divided by calsequestrin levels. Calsequestrin is quantified as a loading control between lanes as is our routine procedure in animal and human cardiac preparations (e.g. Neumann et al. 1998, 2021). We noted that dulaglutide raised both the phosphorylation states of phospholamban at serine 16 (original experiment: Fig. 7A) and the phosphorylation state of the troponin inhibitor (original experiment: Fig. 7B). These data were summed up. We noted that 100 nM dulaglutide in the presence of 100 nM cilostamide raised the phosphorylation of phospholamban significantly compared to 100 nM cilostamide alone (Fig. 7C). Moreover, 100 nM dulaglutide in the presence of 100 nM cilostamide raised the phosphorylation state of the troponin inhibitor significantly compared to 100 nM cilostamide alone (Fig. 7D). Please note that, as expected, the positive control, isoprenaline, augmented both the phosphorylation state of phospholamban and the troponin inhibitor, confirming that we used proper experimental procedures for the contraction experiments and then for the pursuant Western blotting in homogenates from these samples.

## Discussion

The main new findings from this report are that dulaglutide exerted a positive inotropic effect in HAP and that this effect was mediated via GLP-1R and probably involves cAMP-dependent protein phosphorylation in HAP. Hence, the positive inotropic effect of dulaglutide could affect directly human cardiac function and thus may be clinically relevant. These positive inotropic effects of dulaglutide were time- and concentration-dependent in HAP. Dulaglutide was less effective to raise force of contraction than isoprenaline. We have noted this before with exenatide, liraglutide and semaglutide (Neumann et al. 2024). This could be due to the lower number of GLP-1R compared to adrenoceptors in the human heart. However, this assumption is a speculation. As far as we are aware, one has not yet quantified the number and affinity of GLP-1 receptors in the human heart. However, this suggestion is consistent with studies in which the affinity of GLP-1R agonists at transfected human GLP-1R were studied (Sloop et al. 2024). They noticed that GLP-1R were more potent when more receptors was transfected. Hence, low receptor numbers led to less cAMP increase in the cells and this is consistent with our assumption (Sloop et al. 2024). Apparently, the number of GLP-1R in the heart is too low to detect them with ligand binding studies. Moreover, there seem to be no specific antibodies for GLP-1R available in the heart. However, the mRNA of the GLP-1R is present in the human cardiac atrium as others and we have already been reported (Baggio et al. 2017, Baggio et al. 2018; Neumann et al. 2024). One can assume that dulaglutide signals via cAMP (Glaesner et al. 2010). This is consistent with studies in transfected cells where dulaglutide increased cAMP. This is also in agreement with other reports indicating that dulaglutide increased cAMP in cardiac cells. Moreover, our own data with cilostamide point into that direction. Moreover, dulaglutide phosphorylated phospholamban at amino acid serine 16 which is the PKA phosphorylation site (Frank and Kranias 2000, Fig. 1). Dulaglutide half maximally increased the insulin secretion from monkey pancreatic cells at 2.7 nM (Glaesner et al. 2010). Now, one might question why we used cilostamide and not rolipram in HAP and why 100 nM dulaglutide. This is due to our previous studies and those of others. We noted before that 100 nM cilostamide potentiated the positive inotropic effects of exenatide, liraglutide and semaglutide in the isolated human atrium (Neumann et al. 2024) at that concentration. It is further known that cilostamide is a selective phosphodiesterase 3 inhibitor which is the main phosphodiesterase subtype in the human heart (Kamel et al. 2023). In contrast, rolipram does not increase force of contraction in the human but is a potent positive inotropic drug in the mouse heart (Kamel et al. 2023; Neumann et al. 2021). This is due to the fact that rolipram is a phosphodiesterase 4 inhibitor and that phosphodiesterase 4 is highly active in the mouse heart (Kamel et al. 2023). Moreover, we can conclude from the present study that the positive inotropic effects of dulaglutide in the human heart are mediated via GLP1-R because the the positive inotropic effect of dulaglutide were reversed by exendin(9–39), an antagonist at GLP1-R (Serre et al. 1998). These GLP-1R are present in the human atrium and human ventricle (Wallner et al. 2015, Baggio et al. 2017, 2018). GLP-1R are mainly if not exclusively located to the cardiomyocytes in the human heart and not in other cell types like endothelial cells, which is a species difference (McLean et al. 2022). It remains to be studied whether dulaglutide has a positive inotropic effect in the isolated human ventricular muscle. This is currently not well understood. Exenatide and GLP-1 itself only increased force of contraction in about 15% of ventricular human strips but in contrast, exenatide and GLP-1 itself did increase force of contraction in all atrial muscle strips (Wallner et al. 2015). Assuming that the same holds true for dulaglutide, 15% of patients would benefit from ventricular effects of dulaglutide but all patients should benefit from atrial effects of dulaglutide. A better contraction of human atria due to dulaglutide should lead to better filling of the ventricles and hence to better cardiac output in patients with heart failure (Järvinen et al. 1994, Pardaens et al. 1997). But this is speculative. Peak plasma concentrations in humans of dulaglutide were reported as 114 ng/ml (about 2 nM, Geiser et al. 2016) which are lower than the lowest concentration (10 nM) under which we saw an increase in force of contraction in HAPs. It is a central tenet of modern pharmacology that drug effects are concentration dependent. We would argue that if we detect a clear effect at a high concentration of dulaglutide, then it is likely that at lower drug concentrations, an effect on dulaglutide would be expected to exist but with a lower effect size. Hence, in our eyes, it is conceivable but not certain that clinically reached concentrations of dulaglutide affect cardiac function directly in patients. Further studies on concentration dependent effects over time seem mandatory. However, if we had not detected any effects at 100 nM dulaglutide, it would have been very unlikely that an effect at 2 nM dulaglutide could exist. It is further tempting to speculate that the effects of dulaglutide on force of contraction might be smaller in patients suffering from type 2 diabetes. Indeed, five of our patients, suffered from type 2 diabetes (Table 1). However, the response to dulaglutide was not different in this subgroup (data not shown). Here, a further caveat is in order. We did not design this study to detect an influence of type 2 diabetes on the contractile effects of dulaglutide or GLP-1R agonist in general. In addition in such a prospective study there should be good randomisation for sex and the history of the type 2 diabetes. This was beyond the scope of the present pilot study.

We noted that dulaglutide increased the phosphorylation state of phospholamban at serine 16. This is the phosphorylation site of phospholamban by the cAMP-dependent protein kinase. Thus, this result is further evidence that dulaglutide has increased the cAMP-level and then activated the cAMP-dependent protein kinase. These data also explain why dulaglutide raised force of contraction and shortened the time of relaxation (Fig. 1). Likewise, the increase in the phosphorylation state of TnI, we observed here with dulaglutide in HAP can be explained by a mediation via cAMP-dependent protein kinase and should hasten relaxation of HAP (Kentish et al. 2001).

Dulaglutide, like exenatide, liraglutide or semaglutide exerted no positive inotropic effect when given alone in the LA and no positive chronotropic effect in RA (Neumann et al. 2024). The amount of the mRNA coding for GLP-1R was very low in the LA and missing in both the mouse left ventricle and mouse right ventricle (Kim et al. 2013; Ali et al. 2014, Baggio et al. 2017). Hence, the low expression of GLP-1R mRNA in LA might explain why we failed to detect a PIE in the LA. However, cellular heterogeneity might also or alternatively play a role here: GLP1-R seem to be confined to the mouse heart’s endothelial cells in the endocardium (McLean et al. 2022). Thus, it seems reasonable to conclude that dulaglutide failed to increase force of contraction in the left atrium of adult wild type mice. One has to be very clear that the situation might be different in foetal and neonatal mouse cardiomyocytes. This could be studied based on our present work. Now one might interrogate why we failed to detect a positive chronotropic effect in the mouse heart, when the mRNA of the GLP-1R is present in the adult mouse right atrium. Firstly, it might be that the mRNA is not translated to protein, an unlikely explanation. Secondly, the expression might be too low to be of functional relevance. Thirdly, the GLP-1R might be absent from the sinus node. Fourthly the GLP-1R might be present but does not couple in the sinus node to an increase in cAMP. Fifthly, the GLP-1R might simply be lacking in cardiomyocytes and might only be present in endothelial cells, an interpretation we currently favour based on the work of others (McLean et al. 2022). In any case, our data could mean that in mouse cardiac studies, one can assume that any beneficial effects of dulaglutide in cardiac ischemia and reperfusion are not negatively affected by a positive chronotropic effect or positive inotropic effect of dulaglutide and are thus a useful model to study beneficial effects of GLP-1R stimulation of non-cardiomyocytes in the heart. Dulaglutide reduced blood pressure and increased heart rate in patients in clinical studies (Ferdinand et al. 2014). Now, as already mentioned, in contrast to the mouse atria, in the human atria, GLP-1R was mainly, if not exclusively, detected as mRNA in cardiomyocytes and not in endothelial cells (McLean et al. 2022). In situ hybridisation detected mRNA for GLP-1R in human sinoatrial cardiomyocytes, further suggesting a physiological role of the GLP-1R in heart rate regulation not only in mice but also in humans (Baggio et al. 2018). Hence, it is conceivable that the positive inotropic effect of dulaglutide noted in clinical studies is due to a direct stimulation of GLP-1R in the human sinus node (Sanford 2014). This cannot be concluded from our study but remains to be tested in further work using human sinus node cells. This was beyond the scope of the present study.

There are at least two ways to interpret our findings in the mouse atrial preparation. One view would be that the mouse does not recapitulate the effects of dulaglutide in the human heart: in the atria of adult mice (there may well be developmental or disease related other findings) dulaglutide did not increase force. Hence, for instance, atrial fibrillation would never be detected with dulaglutide in the mouse heart and hence it is poor substitute for human studies. A complementary view would be that the mouse exhibits an excellent model to study dulaglutide without any interference from cardiac inotropic cardiac effects. For instance, protective effects of dulaglutide on liver function would not be made difficult to interpret by cardiac side effects.

Dulaglutide was developed by the company Eli Lilly, Indianapolis, Indiana, USA. Our data should be of some clinical interest because dulaglutide is currently heavily used in e.g. the USA: one estimate gives a sales price for dulaglutide of 4.51 billion (American billions) US dollars ($). Dulaglutide is the 63rd most often prescribed drug in the USA (ClinCalc.com).

Limitations: we have not studied human ventricular preparations. There are reports that only in about 15% of patients GLP-1R stimulation by GLP-1 or exenatide led to a positive inotropic effect in isolated human ventricular preparations (Wallner et al. 2015). It may be worth mentioning that these human ventricular preparations were only studied from non-failing hearts (Wallner et al. 2015). More recently, others detected in isolated human ventricular preparations from failing human heart a significant positive inotropic effect to 50 nM semaglutide, another GLP-1R agonist (Krammer et al. 2025). Hence, it would be conceivable that the GLP-1R is more important in the failing than in the non-failing human ventricle which might be tested in future clinical studies. Moreover, we cannot prove and cannot rule out that dulaglutide increases the incidence of atrial arrhythmias in humans. Moreover, dulaglutide leads as we have shown here in principle to cAMP-dependent phosphorylations in HAP, one would strongly assume that also ion channel phosphorylations would occur (which we have not yet studied). This can easily lead to cardiac arrhythmias (Dridi et al. 2020). Moreover, the activity of the Ca2 +/calmodulin dependent protein kinase is lower in murine cardiac ventricle that in human cardiac ventricle (Krammer et al. 2025). We cannot exclude that this may explain or at least contribute to the lack of a positive inotropic effect of dulaglutide in the mouse atrium compared to the human atrium in the present study. An additional contributing factor may lie in the different ratio of the expression of phospholamban and SERCA in the human atrium compared to the mouse atrium (Gombosova et al. 1998; Linck et al. 1996). Moreover, we have not measured if dulaglutide alters Ca^2+^-sparks or the Ca^2+^-leak from the sarcoplasmic reticulum in human atrial cardiomyocytes. On the one hand, we do not have the necessary state of the art equipment (laser scanning microscope) for this type of measurement. On the other hand, for these measurements one needs Ca^2+^- tolerant isolated human atrial cardiomyocytes (e.g. Krammer et al. 2025) which we had not yet established as a method in our lab. We have only spotlighted two substrates for PKA in human atrial preparations. Other laboratories in contrast measured many more substrates. Others even measured more than 100 phosphoproteins after the injection of isoprenaline to living mice. However, we focussed here on the hypothesis that dulaglutide activates PKA. As proof of principle, we concentrated on just two, well-studied substrates (phospholamban and troponin I). In subsequent studies, it might be possible to define other substrates. One must take into account that there are limitations of our current methods. We freeze clamp human atrial preparations with wet weighs around 3 mg. In other studies, 100 mg or more of cardiac tissue was used as starting material for a phosphoproteomics analysis. Others even started with isolated human cardiomyocytes which they skinned before contraction and biochemical analysis (Eisras et al. 2006): This requires less tissue but in skinned fibers one cannot study the function of the GLP-1R because it involves increases in cytosolic Ca^2+^-transients (Krammer et al. 2025; Hegner et al. 2024) which do not exist in skinned fibers.

In summary, dulaglutide heightened inotropy in isolated HAP probably via GLP-1R and cAMP-dependent phosphorylation.

## Data Availability

The data from this study are available from the corresponding author upon reasonable request.

## References

[CR1] Ali S, Ussher JR, Baggio LL, Kabir MG, Charron MJ, Ilkayeva O, Newgard CB, Drucker DJ (2014) Cardiomyocyte glucagon receptor signaling modulates outcomes in mice with experimental myocardial infarction. Mol Metab 4(2):132–143 10.1016/j.molmet.2014.11.005 PMC4314543 25685700

[CR2] Baggio LL, Ussher JR, McLean BA, Cao X, Kabir MG, Mulvihill EE, Mighiu AS, Zhang H, Ludwig A, Seeley RJ, Heximer SP, Drucker DJ (2017) The autonomic nervous system and cardiac GLP-1 receptors control heart rate in mice. Mol Metab 6(11):1339–1349. 10.1016/j.molmet.2017.08.010 PMC5681270 29107282

[CR3] Baggio LL, Yusta B, Mulvihill EE, Cao X, Streutker CJ, Butany J, Cappola TP, Margulies KB, Drucker DJ (2018) GLP-1 receptor expression within the human heart. Endocrinology 159(4):1570–1584. 10.1210/en.2018-00004 PMC5939638 29444223

[CR4] Deng F, Wu W, Fan X, Zhong X, Wang N, Wang Y, Pan T, Du Y (2023Aug) Dulaglutide protects mice against diabetic sarcopenia-mediated muscle injury by inhibiting inflammation and regulating the differentiation of myoblasts. Int J Endocrinol 7(2023):9926462. 10.1155/2023/9926462.PMID:37584041;PMCID:PMC10425251 PMC10425251 37584041

[CR5] Derington CG, Sarwal A, Wei G, Hartsell SE, Throolin M, Singh R, Nevers MR, Zhang C, Katkam N, Takyi A, Chakravartula AR, Babu P, Deshmukh VG, Boucher RE, Drakos SG, Greene T, Shen J, Beddhu S (2025) Liraglutide vs semaglutide vs dulaglutide in veterans with type 2 diabetes. JAMA Netw Open 8(10):e2537297. 10.1001/jamanetworkopen.2025.37297 PMC12519307 41082229

[CR6] Dridi H, Kushnir A, Zalk R, Yuan Q, Melville Z, Marks AR (2020) Intracellular calcium leak in heart failure and atrial fibrillation: a unifying mechanism and therapeutic target. Nat Rev Cardiol 17(11):732–747. 10.1038/s41569-020-0394-8 PMC8362847 32555383

[CR7] Drucker DJ (2022) GLP-1 physiology informs the pharmacotherapy of obesity. Mol Metab 57:101351. 10.1016/j.molmet.2021.101351 PMC8859548 34626851

[CR8] Drucker DJ, Holst JJ (2023) The expanding incretin universe: from basic biology to clinical translation. Diabetologia 66(10):1765–1779. 10.1007/s00125-023-05906-7 36976349

[CR9] Ferdinand KC, White WB, Calhoun DA, Lonn EM, Sager PT, Brunelle R, Jiang HH, Threlkeld RJ, Robertson KE, Geiger MJ (2014) Effects of the once-weekly glucagon-like peptide-1 receptor agonist dulaglutide on ambulatory blood pressure and heart rate in patients with type 2 diabetes mellitus. Hypertension 64(4):731–7. 10.1161/HYPERTENSIONAHA.114.03062 24980665

[CR10] Frank K, Kranias EG (2000Nov) Phospholamban and cardiac contractility. Ann Med 32(8):572–578. 10.3109/07853890008998837 11127935

[CR11] Geiser JS, Heathman MA, Cui X, Martin J, Loghin C, Chien JY, de la Peña A (2016) Clinical pharmacokinetics of dulaglutide in patients with type 2 diabetes: analyses of data from clinical trials. Clin Pharmacokinet 55(5):625–34. 10.1007/s40262-015-0338-3 26507721

[CR12] Gergs U, Neumann J, Simm A, Silber RE, Remmers FO, Läer S (2009) Phosphorylation of phospholamban and troponin I through 5-HT4-receptors in the isolated human atrium. Naunyn Schmiedebergs Arch Pharmacol 379:349–359 10.1007/s00210-008-0371-y 19002436

[CR13] Gergs U, Fahrion CM, Bock P, Fischer M, Wache H, Hauptmann S, Schmitz W, Neumann J (2017) Evidence for a functional role of calsequestrin 2 in mouse atrium. Acta Physiol 219:669–682 10.1111/apha.12766 27484853

[CR14] Glaesner W, Vick AM, Millican R, Ellis B, Tschang SH, Tian Y, Bokvist K, Brenner M, Koester A, Porksen N, Etgen G, Bumol T (2010) Engineering and characterization of the long-acting glucagon-like peptide-1 analogue LY2189265, an Fc fusion protein. Diabetes Metab Res Rev 26(4):287–296. 10.1002/dmrr.1080 20503261

[CR15] Gombosova I, Boknik P, Kirchhefer U, Knapp J, Lüss H, Müller FU, Müller T, Vahlensieck U, Neumann J (1998) Postnatal changes in contractile time parameters, calcium regulatory proteins and phosphatases. Am J Physiol 274:H2123-2132 10.1152/ajpheart.1998.274.6.H2123 9841539

[CR16] Hegner P, Baier MJ, Krammer T, Seitz S, Käs AK, Zschiedrich T, Lukas D, Lutz V, Wolf M, Sinha F, Schopka S, SchmidC, Kozakov K, Provaznik Z, Maier LS, Mustroph J, Wagner S (2024) Semaglutide improves contractile function in human atrialmyocardium of patients with heart failure and preserved ejection fraction. Cardiovasc Res 122(6):675–677. 10.1093/cvr/cvag039 PMC13123652 41632787

[CR17] Järvinen VM, Kupari MM, Hekali PE, Poutanen VP (1994) Right atrial MR imaging studies of cadaveric atrial casts and comparison with right and left atrial volumes and function in healthy subjects. Radiology 191(1):137–42. 10.1148/radiology.191.1.8134560 8134560

[CR18] Jin M, Zhang S, Huang B, Li L, Liang H, Ni A, Han L, Liang P, Liu J, Shi H, Lv P (2024) Dulaglutide treatment reverses depression-like behavior and hippocampal metabolomic homeostasis in mice exposed to chronic mild stress. Brain Behav 14(3):e3448. 10.1002/brb3.3448 PMC10915471 38444330

[CR19] Kamel R, Leroy J, Vandecasteele G, Fischmeister R (2023) Cyclic nucleotide phosphodiesterases as therapeutic targets in cardiac hypertrophy and heart failure. Nat Rev Cardiol 20(2):90–108. 10.1038/s41569-022-00756-z 36050457

[CR20] Kentish JC, McCloskey DT, Layland J, Palmer S, Leiden JM, Martin AF, Solaro RJ (2001) Phosphorylation of troponin I by protein kinase A accelerates relaxation and crossbridge cycle kinetics in mouse ventricular muscle. Circ Res 88(10):1059–65. 10.1161/hh1001.091640 11375276

[CR21] Kim M, Platt MJ, Shibasaki T, Quaggin SE, Backx PH, Seino S, Simpson JA, Drucker DJ (2013) GLP-1 receptor activation and Epac2 link atrial natriuretic peptide secretion to control of blood pressure. Nat Med 19(5):567–75. 10.1038/nm.3128 23542788

[CR22] Krammer T, Baier MJ, Hegner P, Zschiedrich T, Lukas D, Wolf M, Le Phu C, Lutz V, Evert K, Kozakov K, Li J, Holzamer A, Maier LS, Provaznik Z, Bers DM, Wagner S, Mustroph J (2025) Cardioprotective effects of semaglutide on isolated human ventricular myocardium. Eur J Heart Fail 27(7):1315–1325. 10.1002/ejhf.3644 PMC12370581 40107718

[CR23] Kristensen SL, Rørth R, Jhund PS, Docherty KF, Sattar N, Preiss D, Køber L, Petrie MC, McMurray JJV (2019) Cardiovascular, mortality, kidney outcomes with GLP-1 receptor agonists in patients with type 2 diabetes: a systematic review and meta-analysis of cardiovascular outcome trials. Lancet Diabetes Endocrinol 7(10):776–785. 10.1016/S2213-8587(19)30249-9 31422062

[CR24] Kwaah PA, Mensah SA, Carboo A, Rashid HA, Agyemang EA, Appah G, Frimpong SK (2025) Cardiovascular outcomes of semaglutide vs dulaglutide in nonobese type II diabetes patients with HFpEF. JACC Adv 4(7):101857. 10.1016/j.jacadv.2025.101857 PMC12172265 40466245

[CR25] Linck B, Boknik P, Eschenhagen T, Müller FU, Neumann J, Nose M, Jones LR, Schmitz W, Scholz H (1996) Messenger RNA expression and immunological quantification of phospholamban and SR-Ca2+-ATPase in failing and nonfailing human hearts. Cardiovasc Res 31:625–632 8689655

[CR26] Mayo KE, Miller LJ, Bataille D, Dalle S, Göke B, Thorens B, Drucker DJ (2003) International union of pharmacology. XXXV. The glucagon receptor family. Pharmacol Rev 55(1):167–94. 10.1124/pr.55.1.6 12615957

[CR27] McLean BA, Wong CK, Kabir MG, Drucker DJ (2022) Glucagon-like peptide-1 receptor Tie2+ cells are essential for the cardioprotective actions of liraglutide in mice with experimental myocardial infarction. Mol Metab 66:101641. 10.1016/j.molmet.2022.101641 PMC9706177 36396031

[CR28] Neumann J, Boknik P, Bodor GS, Jones LR, Schmitz W, Scholz H (1994) Effects of adenosine receptor and muscarinic cholinergic receptor agonists on cardiac protein phosphorylation. Influence of pertussis toxin. J Pharmacol Exp Ther 269:1310–1318 8014875

[CR29] Neumann J, Boknik P, DePaoli-Roach AA, Field LJ, Rockman HA, Kobayashi YM, Kelley JS, Jones LR (1998) Targeted overexpression of phospholamban to mouse atrium depresses Ca2+ transport and contractility. J Mol Cell Cardiol 30(10):1991–2002. 10.1006/jmcc.1998.0760 9799653

[CR30] Neumann J, Käufler B, Gergs U (2019) Which phosphodiesterase can decrease cardiac effects of 5-HT4 receptor activation in transgenic mice? Naunyn Schmiedebergs Arch Pharmacol 392(8):991–1004. 10.1007/s00210-019-01653-y 31016326

[CR31] Neumann J, Schwarzer D, Fehse C, Schwarz R, Marusakova M, Kirchhefer U, Hofmann B, Gergs U (2021) Functional interaction of H2-receptors and 5HT4-receptors in atrial tissues isolated from double transgenic mice and from human patients. Naunyn Schmiedebergs Arch Pharmacol 394(12):2401–2418. 10.1007/s00210-021-02145-8 PMC8592968 34562141

[CR32] Neumann J, Hadova K, Klimas J, Hofmann B, Gergs U (2024) Contractile effects of semaglutide in the human atrium. Pharmaceutics 16(9):1139. 10.3390/pharmaceutics16091139 PMC11435389 39339176

[CR33] Pardaens K, Van Cleemput J, Vanhaecke J, Fagard RH (1997) Atrial fibrillation is associated with a lower exercise capacity in male chronic heart failure patients. Heart 78(6):564–8. 10.1136/hrt.78.6.564 PMC1892336 9470871

[CR34] Sanford M (2014) Dulaglutide: first global approval. Drugs 74(17):2097–103. 10.1007/s40265-014-0320-7 25367716

[CR35] Serre V, Dolci W, Schaerer E, Scrocchi L, Drucker D, Efrat S, Thorens B (1998Nov) Exendin-(9–39) is an inverse agonist of the murine glucagon-like peptide-1 receptor: implications for basal intracellular cyclic adenosine 3’,5’-monophosphate levels and beta-cell glucose competence. Endocrinology 139(11):4448–4454. 10.1210/endo.139.11.6295 9794451

[CR36] Sloop KW, Cox AL, Wainscott DB, White A, Droz BA, Stutsman C, Showalter AD, Suter TM, Dunbar JD, Snider BM, O’Farrell LS, Hewitt N, Ruble JC, Padgett LR, Woerly EM, Peterson JA, Coskun T, Liu Z, Coutant DE, Ai M, Emmerson PJ, Sangwung P, Willard FS (2024) The pharmacological basis for nonpeptide agonism of the GLP-1 receptor by orforglipron. Sci Transl Med 16(778):eadp5765. 10.1126/scitranslmed.adp5765 39693407

[CR37] Wallner M, Kolesnik E, Ablasser K, Khafaga M, Wakula P, Ljubojevic S, Thon-Gutschi EM, Sourij H, Kapl M, Edmunds NJ, Kuzmiski JB, Griffith DA, Knez I, Pieske B, von Lewinski D (2015Dec) Exenatide exerts a PKA-dependent positive inotropic effect in human atrial myocardium: GLP-1R mediated effects in human myocardium. J Mol Cell Cardiol 89(Pt B):365–375. 10.1016/j.yjmcc.2015.09.018 26432951

[CR38] Wegener AD, Jones LR (1984 Feb 10) Phosphorylation-induced mobility shift in phospholamban in sodium dodecyl sulfate-polyacrylamide gels. Evidence for a protein structure consisting of multiple identical phosphorylatable subunits. J Biol Chem 259(3):1834–41 6229539

[CR39] Xie S, Zhang M, Shi W, Xing Y, Huang Y, Fang WX, Liu SQ, Chen MY, Zhang T, Chen S, Zeng X, Wang S, Deng W, Tang Q (2022) Long-term activation of glucagon-like peptide-1 receptor by dulaglutide prevents diabetic heart failure and metabolic remodeling in type 2 diabetes. J Am Heart Assoc 11(19):e026728. 10.1161/JAHA.122.026728 PMC9673690 36172969

